# Escaping The Curse of Dimensionality in Bayesian Model-Based Clustering

**Published:** 2023-04

**Authors:** Noirrit Kiran Chandra, Antonio Canale, David B. Dunson

**Affiliations:** Department of Mathematical Sciences The University of Texas at Dallas Richardson, TX, USA; Department of Statistical Sciences University of Padova Padova, Italy; Department of Statistical Science Durham, NC, USA

**Keywords:** Big data, Clustering, Dirichlet process, Exchangeable partition probability function, High dimensional, Latent variables, Mixture model

## Abstract

Bayesian mixture models are widely used for clustering of high-dimensional data with appropriate uncertainty quantification. However, as the dimension of the observations increases, posterior inference often tends to favor too many or too few clusters. This article explains this behavior by studying the random partition posterior in a non-standard setting with a fixed sample size and increasing data dimensionality. We provide conditions under which the finite sample posterior tends to either assign every observation to a different cluster or all observations to the same cluster as the dimension grows. Interestingly, the conditions do not depend on the choice of clustering prior, as long as all possible partitions of observations into clusters have positive prior probabilities, and hold irrespective of the true data-generating model. We then propose a class of latent mixtures for Bayesian clustering (Lamb) on a set of low-dimensional latent variables inducing a partition on the observed data. The model is amenable to scalable posterior inference and we show that it can avoid the pitfalls of high-dimensionality under mild assumptions. The proposed approach is shown to have good performance in simulation studies and an application to inferring cell types based on scRNAseq.

## 1. Introduction

High-dimensional data $y_{i}={\left(y_{i1},\ldots ,y_{ip}\right)}^{\text{T}}$ for i=1,…,n, with p≫n, have become commonplace, and there is routinely interest in clustering observations {1,…,n} into groups. As an illustrative application, we consider single-cell RNA sequencing (scRNASeq) data; clustering of the cells based on their high-dimensional gene expression profiles produces potential cell types and provides information on heterogeneous cell populations of potential utility in disentangling carcinogenic processes. RNAseq data is an exemplary setting in which p is massive and clustering is crucial due to interest in inferring cell types. Although there are a variety of alternatives in the literature (see Kiselev et al., 2019, for a review), we are particularly motivated to consider a Bayesian approach due to the potential for propagating uncertainty in inferring cell types. Additionally hierarchical Bayes models allow for borrowing of information in a principled manner in complicated scenarios.

Bayesian clustering is typically based on mixture models of the form:

(1) $$y_{i}\overset{\text{iid}}{∼}f,\;f(y)=\sum_{h=1}^{k}\;\pi_{h}K\left(y;\theta_{h}\right),$$

where f(⋅) is the marginal density of the data, k is the number of components, $\pi ={\left(\pi_{1},\ldots ,\pi_{k}\right)}^{\text{T}}$ are probability weights, $K\left(y;\theta_{h}\right)$ is the density of the data within component h, and the number of clusters in data $y_{1},\ldots ,y_{n}$ corresponds to the number of occupied components $k_{n}\leq k$. When p is large and $y_{i}\in {\mathbb{R}}^{p}$, a typical approach chooses $K\left(y;\theta_{h}\right)$ as a multivariate Gaussian density with a constrained and parsimonious covariance (see Bouveyron and Brunet-Saumard, 2014, for a review). Examples include matrices that are diagonal (Banfield and Raftery, 1993), block diagonal (Galimberti and Soffritti, 2013) or have a factor analytic representation (Ghahramani et al., 1996).

To avoid sensitivity to a pre-specified k, one can place a prior on k to induce a mixture of finite mixture model (Miller and Harrison, 2018; Frühwirth-Schnatter et al., 2021). Alternatively, a Bayesian nonparametric approach lets k=∞, which allows $k_{n}$ to increase without a bound as n increases. Under a Dirichlet process (Ferguson, 1973) $k_{n}$ increases at a log rate in n, while for a Pitman-Yor process (Pitman and Yor, 1997) the rate is a power law.

Notably, Bayesian approaches can be used to intrinsically regularize the model complexity, as discussed by Jefferys and Berger (1992) exploiting the idea of a ‘Bayesian Ockham razor’. While in many circumstances relying on the Bayesian Ockham razor is sufficient to choose the appropriate compromise between extremes, e.g. too many or too few clusters, in what follows we will argue that this is not the case in high-dimensional clustering. Indeed, when p is very large, the posterior distribution of $k_{n}$ can concentrate on large values (Celeux et al., 2018); often the posterior mode of $k_{n}$ is even equal to *n* so that each subject is assigned to its own singleton cluster. Consider, for example, the right panel of Figure S.1 in the supplementary materials, which displays the distribution of the mean number of clusters in 100 replicates of a simple simulation example where we generate samples of size n=10 from a p=20 variate normal distribution with mean zero and identity covariance. The boxplot, obtained running a standard Dirichlet process mixture, clearly shows how $k_{n}$ is concentrated near n even for this moderate value of p. Celeux et al. (2018) conjectured that this aberrant behavior is mainly due to slow mixing of Markov chain Monte Carlo samplers. Frühwirth-Schnatter (2006) combat this problem with a specific prior elicitation criterion; this can be successful for p≈100, but calibration of hyperparameters is a delicate issue and scaling to p>1,000 is problematic. Alternatively, one may attempt to cluster in lower dimensions via variable selection in clustering (Tadesse et al., 2005; Kim et al., 2006) or by introducing both global and variable-specific clustering indices for each subject, so that only certain variables inform global cluster allocation (Dunson, 2009).

However, we find these approaches complicated and to not address the fundamental question of what is causing the poor performance of Bayesian clustering for large p. To fill this gap, we provide theory showing that, as p→∞ with n fixed, the posterior can assign probability one to a trivial clustering - either with $k_{n}=1$ and all subjects in one cluster or with $k_{n}=n$ and every subject in a different cluster. We further show that the conditions under which these degenerate limiting behaviors occur are satisfied for seemingly standard priors and multivariate Gaussian kernels. In a related result for classification, Bickel and Levina (2004) showed that when p increases at a faster rate than n, the Fisher’s linear discriminant rule is equivalent to randomly assigning future observations to the existing classes.

Our result has no relationship with the literature studying the posterior behavior of $k_{n}$ as n→∞ for nonparametric Bayes procedures (Miller and Harrison, 2014; Cai et al., 2020; Ascolani et al., 2022). Indeed, our result holds for finite n regardless of the true data generating model, and has fundamentally different implications—in particular, that one needs to be extremely careful in specifying the kernel K(y;θ) and prior for θ in the large p context. Otherwise, the true posterior can produce degenerate clustering results that have nothing to do with true structure in the data.

A key question is whether it is possible to define models that can circumvent this pitfall? We show that the answer is yes if clustering is conducted on the level of low-dimensional latent variables $\eta_{i}$ underlying $y_{i}$. When the dimension of $\eta_{i}$ is small relative to p, $y_{i}$ provides abundant information about the lower-dimensional $\eta_{i}$ even in low signal-to-noise settings in which each individual $y_{ij}$ contributes very little information on its own. Hence, the curse of dimensionality can be turned into a blessing. This motivates a novel notion of a Bayesian oracle for clustering. The oracle has knowledge of the latent $\eta_{i}\text{s}$ and defines a Bayesian mixture model for clustering based on the $\eta_{i}\text{s}$; the resulting oracle clustering posterior is thus free of the curse of dimensionality. We propose a particular latent mixture model structure, which can be shown to satisfy this oracle property and additionally leads to straightforward computation.

The article is organized as follows. Section 2 gives details on the limiting behavior of usual clustering methods based on (1). Section 3 introduces our mixture model on the latent variable level with prior specifications and posterior computation strategies. In Section 4, we introduce a Bayesian oracle clustering rule and show that our model achieves this oracle property as the dimension grows to infinity. Section 5 shows simulation studies illustrating how our proposed model learns the latent space with increasing dimensions and compares our method with some popular clustering methods. Section 6 considers an application to scRNASeq data, and Section 7 discusses the results. Proofs of the main results are included in the Appendix while additional technical results, simulation studies, and MCMC convergence diagnostics are reported in the supplementary materials.

## 2. Limiting Behavior of High-Dimensional Bayesian Clustering

Under a general Bayesian framework, model (1) becomes

(2) $$y_{i}∼f,\;f(y)=\sum_{h\geq 1}\pi_{h}K\left(y;\theta_{h}\right),\;\theta_{h}\overset{\text{iid}}{∼}P_{0},\;\left\{\pi_{h}\right\}∼Q_{0},$$

where $\left\{\pi_{h}\right\}∼Q_{0}$ denotes a suitable prior for the mixture weights. Examples include stick-breaking (Sethuraman, 1994) constructions or a k-dimensional Dirichlet distribution with the dimension k given a prior following a mixture of finite mixtures (MFMs) approach.

Let $c_{i}\in \{1,\ldots ,\infty \}$ denote the cluster label for subject i (for i=1,…,n), with $k_{n}=\text{\#}\left\{c_{1},\ldots ,c_{n}\right\}$ denoting the number of clusters represented in the sample. Conditionally on $c_{i}=h$, we can write $y_{i}|c_{i}=h∼K\left(y_{i};\theta_{h}\right)$. Assume that $n_{j}$ is the size of the jth cluster with $\sum_{i=1}^{k}\;n_{j}=n$. The posterior probability of observing the partition Ψ induced by the clusters $c_{1},\ldots ,c_{n}$ conditionally on the data $Y=\left\{y_{1},\ldots ,y_{n}\right\}$ is

(3) $$\text{Π}(\text{Ψ}|Y)=\frac{\text{Π}(\text{Ψ})\times \prod_{h\geq 1}\int \prod_{i:c_{i}=h}K\left(y_{i};\theta \right)\text{d}P_{0}(\theta )}{\sum_{{\text{Ψ}}^{\prime }\in P}\text{Π}\left({\text{Ψ}}^{\prime }\right)\times \prod_{h\geq 1}\int \prod_{i:c_{i}=h}K\left(y_{i};\theta \right)\text{d}P_{0}(\theta )},$$

where P is the space of all possible partitions of n data points into clusters. The numerator of (3) is the product of the prior probability of Ψ multiplied by a product of the marginal likelihoods of the observations within each cluster. The denominator is a normalizing constant consisting of an enormous sum over P. Assuming exchangeability, the prior probability of any partition of n subjects into $k_{n}$ groups depends only on $n_{1},\ldots ,n_{k_{n}}$ and $k_{n}$ through an exchangeable partition probability function (EPPF). The latter is available in closed form for popular choices of $Q_{0}$, including the Dirichlet process, Pitman-Yor process and certain MFMs.

The posterior (3) forms the basis for Bayesian inferences on clusterings in the data, while providing a characterization of uncertainty. We are particularly interested in how this posterior behaves in the case in which $y_{i}={\left(y_{i1},\ldots ,y_{ip}\right)}^{\text{T}}$ are high-dimensional so that p is very large. To study this behavior theoretically, we consider the limiting case as p→∞ while keeping n fixed. This setting is quite appropriate in our motivating applications to genomics, as there is essentially no limit to the number of variables one can measure on each study subject, while the number of study subjects is often small to moderate.

In such settings with enormous p and modest n, we would like the true posterior distribution in (3) to provide a realistic characterization of clusters in the data. However, this is commonly not the case and as p increases the posterior distribution can have one of two trivial degenerate limits. In particular, depending on the choice of kernel density K(⋅;θ) and the base measure $P_{0}$ for the $\theta_{h}$’s, the posterior assigns probability one to either the $k_{n}=1$ clustering that places all subjects in the same cluster or the $k_{n}=n$ clustering that places all subjects in different clusters. We derive sufficient conditions behind such aberrant behaviors as formalized in the following theorem.

### Theorem 1

*Let* $y_{1},\ldots ,y_{n}$ *denote p-variate random vectors with joint probability measure* ${\mathbb{P}}_{0}^{p}$. *Let* Ψ *denote the partition induced by the cluster labels* $c_{1},\ldots ,c_{n}$, *and let* $c_{1}^{\prime },\ldots ,c_{n}^{\prime }$ *denote a new set of cluster labels obtained from* $c_{1},\ldots ,c_{n}$ *by merging an arbitrary pair of clusters, with* ${\text{Ψ}}^{\prime }$ *the related partition*. *Assume* $Q_{0}\left(\pi_{h}>0\;for\;all\;h=1,\ldots ,n\right)>0$. *If*

$$\underset{p\to \infty }{\lim\;\sup}\;\frac{\prod_{h\geq 1}\int \prod_{i:c_{i}=h}K\left(y_{i};\theta \right)\text{d}P_{0}(\theta )}{\prod_{h\geq 1}\int \prod_{i:c_{i}^{\prime }=h}K\left(y_{i};\theta \right)\text{d}P_{0}(\theta )}=0$$

*in* ${\mathbb{P}}_{0}^{p}$-*probability, then* $\lim_{p\to \infty }\;\text{Π}\left(c_{1}=\cdots =c_{n}|Y\right)=1$ *in* ${\mathbb{P}}_{0}^{p}$-*probability*. *Else if*

$$\underset{p\to \infty }{\lim\;\text{inf}}\;\frac{\prod_{h\geq 1}\int \prod_{i:c_{i}=h}K\left(y_{i};\theta \right)\text{d}P_{0}(\theta )}{\prod_{h\geq 1}\int \prod_{i:c_{i}^{\prime }=h}K\left(y_{i};\theta \right)\text{d}P_{0}(\theta )}=\infty$$

*in* ${\mathbb{P}}_{0}^{p}$-*probability, then* $\lim_{p\to \infty }\;\text{Π}\left(c_{1}\neq \cdots \neq c_{n}|Y\right)=1$ *in* ${\mathbb{P}}_{0}^{p}$-*probability*.

The condition on $Q_{0}$ is equivalent to saying $k_{n}$ has positive prior mass on 1,…,n, which is extremely mild and holds for essentially any prior in the literature, including the Dirichlet process, Pitman-Yor process and suitable MFMs that do not pre-specify k<n. Changing the condition to $Q_{0}\left(\pi_{h}>0\;\text{for all}\;h=1,\ldots ,k\right)>0$ with k<n, i.e. using a finite mixture model, leads to similar results. Specifically, if the first condition in Theorem 1 holds, then also for finite mixtures we will have a single occupied cluster comprising all samples. If the opposite condition holds, instead, then all of the k mixture components will be occupied. Both results are trivial modifications of the proof of Theorem 1.

Theorem 1 has disturbing implications in terms of the behavior of posterior distributions for Bayesian clustering in large p settings. Notably, the theorem is stated for very general kernel density K and base measure $P_{0}$, and the behavior is controlled by the induced marginal likelihoods obtained in integrating out the kernel parameter θ with respect to $P_{0}$. Clearly it is the joint effect of K and $P_{0}$ that leads to the two limiting results and thus it is not immediate to convert the statement of the theorem to simple conditions on K and $P_{0}$. However, as we will discuss in detail, we can argue that these conditions are related to the two extreme situations of complex over-parametrized models having insufficiently informative priors and simpler models equipped with more informative priors. To be more precise, consider the important and widely used special case corresponding to a location-scale mixture of multivariate Gaussian kernels:

(4) $$y_{i}\overset{\text{iid}}{∼}f,\;f(y)=\sum_{h\geq 1}\pi_{h}{\text{N}}_{p}\left(y;\mu_{h},{\text{Σ}}_{h}\right),\;\left(\mu_{h},{\text{Σ}}_{h}\right)\overset{\text{iid}}{∼}P_{0},$$

where ${\text{N}}_{p}(\mu ,\text{Σ})$ denotes the p-dimensional multivariate normal density with mean μ and covariance matrix Σ. We give two practical examples of Theorem 1 in Corollary 2 and 3. Let $\lambda_{\min}(A)$ and $\lambda_{\max}(A)$ be the smallest and largest eigenvalues of a positive definite matrix A and $Y={\left[y_{1},\ldots ,y_{n}\right]}^{\text{T}}$ be the complete n×p data matrix. Assume, for the true data generating distribution on the data Y,

(A0) ${\lim\;\text{inf}}_{p\to \infty }\;\lambda_{\min}\left(YY^{\text{T}}\right)/p>0$ in ${\mathbb{P}}_{0}^{p}$-probability and ${\left\|y_{i}\right\|}_{2}\leq Kp$ for some K>0 in ${\mathbb{P}}_{0}^{p}$-probability.

Condition (A0) is extremely mild ensuring that the data are non-atomic and is satisfied for any continuous distribution with finite second order moments. Letting IW(ν,Λ) denote an inverse-Wishart distribution with degrees of freedom ν and scale matrix Λ, we have the following:

### Corollary 2

*Assume that the model* (4) *is used to cluster* Y *with* ${\text{Σ}}_{h}\overset{\text{iid}}{∼}\text{IW}\left(\nu_{0},{\text{Λ}}_{0}\right)$ *and* $\mu^{h}|{\text{Σ}}_{h}\overset{\text{ind}}{∼}{\text{N}}_{p}\left(\mu_{0},\kappa_{0}^{-1}{\text{Σ}}_{h}\right)$, *with* ${\left\|\mu_{0}\right\|}_{2}=O(p)$, $\kappa_{0}=O(1)$, $\nu_{0}=p+c$ *for some fixed constant* c≥0, ${\left\|{\text{Λ}}_{0}\right\|}_{2}=O(1)$ *and* ${\left\|{\text{Λ}}_{0}\right\|}_{2}/\lambda_{\min}\left({\text{Λ}}_{0}\right)=O(1)$. *Under (A0) on the data* Y, $\text{Π}\left(c_{1}=\cdots =c_{n}|Y\right)\to 1$ *in* ${\mathbb{P}}_{0}^{p}$-*probability*.

### Corollary 3

*Assume that the model* (4) *is used to cluster* Y *with* ${\text{Σ}}_{h}=\text{Σ}$ *across all clusters, and let* $\text{Σ}∼\text{IW}\left(\nu_{0},{\text{Λ}}_{0}\right)$ *and* $\mu_{h}|\text{Σ}\overset{\text{iid}}{∼}{\text{N}}_{p}\left(\mu_{0},\kappa_{0}^{-1}\text{Σ}\right)$, *with* ${\left\|\mu_{0}\right\|}^{2}=O(p)$, $\kappa_{0}=O(1)$, $\nu_{0}>p-1$ *such that* $\lim_{p\to \infty }\;\nu_{0}/p>1$, *and* ${\left\|{\text{Λ}}_{0}\right\|}_{2}=O(1)$ *with* ${\left\|{\text{Λ}}_{0}\right\|}_{2}/\lambda_{\min}\left({\text{Λ}}_{0}\right)=O(1)$. *Under (A0) on the data* Y, $\text{Π}\left(c_{1}\neq \cdots \neq c_{n}|Y\right)\to 1$ *in* ${\mathbb{P}}_{0}^{p}$-*probability*.

Bayesian model-based clustering routinely uses these setups for the kernel parameters and priors (Fruhwirth-Schnatter et al., 2019). The conditions on $\mu_{0}$ and $\kappa_{0}$ ensure that the Euclidean norm of the prior mean grows with p in the same order as the data $\left\{y_{i}\right\}$, and the conditions on the scale matrix ${\text{Λ}}_{0}$ imply that the second moments of the location components are a priori bounded away from 0 while being finite; similar assumptions appear in Yao et al. (2022) in a study on high-dimensional Gaussian location mixture models. In terms of the degrees of freedom parameter $\nu_{0}$, in Corollary 2 the ratio $\nu_{0}/p$ is 1 in the limit inducing a heavy tailed prior predictive distribution, whereas in Corollary 3 a thinner tailed prior predictive is induced. Corollaries 2 and 3 show that, for mixtures of Gaussians, we can obtain directly opposite aberrant limiting behavior of the posterior depending on the kernel and prior for the kernel parameters but not on the clustering prior $Q_{0}$.

Corollary 2 considers the case in which we allow flexible cluster-specific means and dispersion matrices, under typical conjugate multivariate normal IW priors. This case can be viewed as a complex over-parametrized model as p increases and to combat this complexity the Bayesian Ockham razor (Jefferys and Berger, 1992) automatically assigns probability one to grouping all n individuals into the same cluster effectively simplifying the model. At the other extreme, covered by Corollary 3, we assume an under-parametrized relatively simplistic model structure in which all the mixture components have a common covariance. In this case, due perhaps to the relatively concentrated prior predictive distribution, there is not enough penalty for introducing new clusters, and all individuals are assigned to their own singleton cluster. These results hold regardless of the true data-generating model, and in particular the true clustering structure.

These theoretical results demonstrate that in high dimensions it is crucial to choose a good compromise between parsimony and flexibility in Bayesian model-based clustering. Otherwise, the true posterior distribution of clusterings in the data can have effectively no relationship whatsoever with true clustering structure in the data. Although we focus on the limiting case as p→∞, we conjecture that this behavior can ‘kick in’ quickly as p increases, based on intuition built through our proofs and through comprehensive simulation experiments.

## 3. Latent Factor Mixture

To overcome the problems discussed in Section 2, we propose a general class of latent factor mixture models defined as

(5) $$y_{i}∼f\left(y_{i};\eta_{i},\psi \right),\;\eta_{i}∼\sum_{h=1}^{\infty }\;\pi_{h}K\left(\eta_{i};\theta_{h}\right),$$

where $\eta_{i}={\left(\eta_{i1},\ldots ,\eta_{id}\right)}^{\text{T}}$ are d-dimensional latent variables, d<n is fixed and not growing with p, $f\left(\cdot ;\eta_{i},\psi \right)$ is the density of the observed data conditional on the latent variables and measurement parameters ψ and K(⋅;θ) is a d-dimensional kernel density.

Under (5), the high dimensional data being collected are assumed to provide error-prone measurements of an unobserved lower-dimensional set of latent variables $\eta_{i}$ on subject i. As a canonical example, we focus on a linear Gaussian measurement model with a mixture of Gaussians for the latent factors:

(6) $$y_{i}∼{\text{N}}_{p}\left(\text{Λ}\eta_{i},\text{Σ}\right),\;\eta_{i}∼\sum_{h=1}^{\infty }\;\pi_{h}{\text{N}}_{d}\left(\mu_{h},{\text{Δ}}_{h}\right),\;\left\{\pi_{h}\right\}∼Q_{0},$$

where $\text{Σ}=\text{diag}\left(\sigma_{1}^{2},\ldots ,\sigma_{p}^{2}\right)$ is a p×p diagonal matrix, and Λ is a p×d matrix of factor loadings. The key idea is to incorporate all the cluster-specific parameters at the latent data level instead of the observed data level to favor parsimony. The latent variables are supported on a lower-dimensional hyperplane, and we map from this hyperplane to the observed data level through multiplication by a factor loadings matrix and then adding Gaussian noise. We could further simplify the model by assuming $\text{Σ}=\sigma^{2}I_{p}$ instead of Σ diagonal; we find it appealing to allow the different $y_{ij}\text{s}$ to have varying measurement error variances and hence focus mainly on the unconstrained diagonal case. We refer to model (6) as a LAtent Mixture for Bayesian (Lamb) clustering. The model is highly flexible at the latent variable level, allowing differences across clusters in the mean through $\mu_{h}$ and the shape, size, and orientation through ${\text{Δ}}_{h}$.

With different motivations, Galimberti et al. (2009); Baek et al. (2010); Montanari and Viroli (2010) proposed similar latent factor mixture models as (6) albeit with additional constraints. Moreover, they fixed the number of clusters, used EM algorithms for model fitting and assessed goodness-of-fit via information criteria.

The proposed Lamb model has fundamentally different implications from the popular mixture of factor analyzers of Ghahramani et al. (1996), which defines a mixture of multivariate Gaussians at the p-dimensional observed data level having cluster-specific means and covariance matrices, with the dimension of the covariances reduced via a factor model. In contrast, we are effectively learning a common affine space within which we can define a simple location-scale mixture of Gaussians. Our approach not only massively reduces the effective number of parameters for large p, but also provides a successful compromise between the two extreme cases of Section 2.

### 3.1 Prior Specifications

In order to accommodate very high-dimensional data, with p≫n, it is important to reduce the effective number of parameters in the p×d loadings matrix Λ. There is a rich literature on sparse factor modeling using a variety of shrinkage or sparsity priors for Λ; for example, refer to Bhattacharya and Dunson (2011) and the references therein. Although a wide variety of shrinkage priors for Λ are appropriate, we focus on a Dirichlet-Laplace prior (Bhattacharya et al., 2015), as it is convenient both computationally and theoretically. On a p-dimensional vector θ, the Dirichlet-Laplace prior with parameter a, denoted by DL(a), can be specified in the following hierarchical manner

(7) $$\theta_{j}|\phi ,\tau \overset{\text{ind}}{∼}\text{N}\left(0,\psi_{j}\phi_{j}^{2}\tau^{2}\right),\;\psi_{j}\overset{\text{iid}}{∼}\text{Exp}(1/2),\;\phi ∼\text{Dir}(a,\ldots ,a),\;\tau ∼\text{Ga}(pa,1/2),$$

where $\theta_{j}$ is the j-th element of θ, ϕ is a vector of the same length as θ, Exp(a) is an exponential distribution with mean 1/a, $\text{Dir}\left(a_{1},\ldots ,a_{p}\right)$ is the p-dimensional Dirichlet distribution, and Ga(a,b) is the gamma distribution with mean a/b and variance $a/b^{2}$. To impose shrinkage uniformly on its elements a priori, we let vec(Λ)∼DL(a) where vec(Λ) denotes the vectorization of Λ. We then choose inverse-gamma priors for the residual variances, $\sigma_{j}^{-2}\overset{\text{iid}}{∼}\text{Ga}\left(a_{\sigma },b_{\sigma }\right)$.

For the prior $Q_{0}$ on the cluster weights $\left\{\pi_{h}\right\}$, for convenience in computation, we use a stick-breaking prior (Ishwaran and James, 2001) derived from a Dirichlet process, which has concentration parameter α impacting the induced prior on the number of clusters. To allow greater data adaptivity, we choose a $\text{Ga}\left(a_{\alpha },b_{\alpha }\right)$ prior for α. We assign the cluster-specific means and covariances {$\mu_{h}$, ${\text{Δ}}_{h}$} independent multivariate normal inverse-Wishart priors with location $\mu_{0}$, precision parameter $\kappa_{0}$, inverse scale matrix ${\text{Δ}}_{0}$ and degrees of freedom $\nu_{0}$. Our hierarchical Bayesian model for the $\eta_{i}\text{s}$ can be equivalently represented as

(8) $$\eta_{i}\left|\mu_{i},{\text{Δ}}_{i}\overset{\text{ind}}{∼}{\text{N}}_{d}\left(\mu_{i},{\text{Δ}}_{i}\right),\;\mu_{i},{\text{Δ}}_{i}\right|G\overset{\text{iid}}{∼}G,\;G∼\text{DP}\left(\alpha ,G_{0}\right),\;\alpha ∼\text{Ga}\left(a_{\alpha },b_{\alpha }\right),$$

where $G_{0}=\text{NIW}\left(\mu_{0},{\text{Δ}}_{0},\kappa_{0},\nu_{0}\right)$. The gamma prior on the concentration parameter α is commonly adopted in many applications motivated by Escobar and West (1995). The role of this hyperprior and the elicitation of its hyperparameters has been carefully studied by Frühwirth-Schnatter and Malsiner-Walli (2019), and Ascolani et al. (2022) recently showed the prior to have a crucial impact on consistency in estimating the number of clusters.

In practice, the latent variable dimension d is unknown. Potentially we could put a prior on d and implement a reversible-jump type (Richardson and Green, 1997) Markov chain Monte Carlo (MCMC) algorithm, which may lead to inefficient and expensive computation. Instead we adopt a principal component analysis (PCA) based empirical Bayes type approach (Bai and Ng, 2008) to set d to a large value learned from the data and let the prior shrink the extra columns on Λ. We use the augmented implicitly restarted Lanczos bidiagonalization algorithm (Baglama and Reichel, 2005) to obtain approximate singular values and eigenvectors, and choose the smallest $\hat{d}$ explaining at least 95% of the variability in the data. This strategy substantially simplifies the computation. The left and right singular values are used to initialize the Λ and $\eta_{i}$’s in our MCMC implementation. We initialize our cluster membership indicators using k-means.

For all the simulation experiments of the next section and the application, we choose $\mu_{0}=0$ and ${\text{Δ}}_{0}=\xi^{2}I_{d}$ for a scalar $\xi^{2}>0$. To specify weakly informative priors, we set $\xi^{2}=20$, $\kappa_{0}=0.001$, $\nu_{0}=\hat{d}+50$, $a_{\alpha }=b_{\alpha }=0.1$ as the hyper-parameters of the DP mixture prior; $a_{\sigma }=1$, $b_{\sigma }=0.3$ as the hyper-parameters of the prior on the residual variances. We set a=0.5 as the Dirichlet-Laplace parameter following the recommendation of Bhattacharya et al. (2015).

### 3.2 Posterior Sampling

For posterior computation we use a Gibbs sampler defined by the following steps.

#### Step 1

Letting $\lambda_{j}^{\text{T}}$ denote the jth row of Λ, $\eta ={\left[\eta_{1},\ldots ,\eta_{n}\right]}^{\text{T}}$, $D_{j}=\tau^{2}\text{diag}\left(\psi_{j1}\phi_{j1}^{2},\ldots ,\psi_{jd}\phi_{jd}^{2}\right)$ and $y^{\left(j\right)}={\left(y_{1j},\ldots ,y_{nj}\right)}^{\text{T}}$, for j=1,…,p sample

$$\left(\lambda_{j}|-\right)∼{\text{N}}_{d}\left\{{\left(D_{j}^{-1}+\sigma_{j}^{-2}\eta^{\text{T}}\eta \right)}^{-1}\eta^{\text{T}}\sigma_{j}^{-2}y^{\left(j\right)},{\left(D_{j}^{-1}+\sigma_{j}^{-2}\eta^{\text{T}}\eta \right)}^{-1}\right\}.$$

#### Step 2

Update the ${\text{Δ}}_{h}$’s from the inverse-Wishart distributions $\text{IW}\left({\hat{\psi }}_{h},{\hat{\nu }}_{h}\right)$ where

$${\bar{\eta }}_{h}=\frac{1}{n_{h}}\sum_{i:c_{i}=h}\eta_{i},\;{\hat{\nu }}_{h}=\nu_{0}+n_{h},\;\;{\hat{\psi }}_{h}=\xi^{2}I_{d}+\sum_{i:c_{i}=h}\left(\eta_{i}-{\bar{\eta }}_{h}\right){\left(\eta_{i}-{\bar{\eta }}_{h}\right)}^{\text{T}}+\frac{\kappa_{0}n_{h}}{\kappa_{0}+n_{h}}{\bar{\eta }}_{h}{\bar{\eta }}_{h}^{\text{T}}.$$

Due to conjugacy, the location parameters $\mu_{h}$’s can be integrated out of the model.

#### Step 3

Sample the latent factors, for i=1,…,n, from

$$\left(\eta_{i}|-\right)∼{\text{N}}_{d}\left\{{\text{Ω}}_{h}\rho_{h},{\text{Ω}}_{h}+{\text{Ω}}_{h}{\left({\hat{\kappa }}_{h,-i}{\text{Δ}}_{h}\right)}^{-1}{\text{Ω}}_{h}\right\},$$

where $n_{h,-i}=\sum_{j\neq i}𝟙\left(c_{j}=h\right)$, ${\hat{\kappa }}_{h,-i}=\kappa_{0}+n_{h,-i}$, ${\bar{\eta }}_{h,-i}=\frac{1}{n_{h,-i}}\sum_{j:c_{j}=h,j\neq i}\eta_{i}$, ${\hat{\mu }}_{h,-i}=\frac{n_{h,-i}{\bar{\eta }}_{h,-i}}{n_{h,-i}+\kappa_{0}}$, $\rho_{h}={\text{Λ}}^{\text{T}}{\text{Σ}}^{-1}Y_{i}+{\text{Δ}}_{h}^{-1}{\hat{\mu }}_{h,-i}$ and ${\text{Ω}}_{h}^{-1}={\text{Λ}}^{\text{T}}{\text{Σ}}^{-1}\text{Λ}+{\text{Δ}}_{h}^{-1}$.

#### Step 4

Sample the cluster indicator variables $c_{1},\ldots ,c_{n}$ with probabilities

(9) $$\text{Π}\left(c_{i}=h|-\right)\propto \{\begin{array}{l} n_{h,-i}\int {\text{N}}_{d}\left(\eta_{i};\mu_{h},{\text{Δ}}_{h}\right)\text{dΠ}\left(\mu_{h},{\text{Δ}}_{h}|c_{-i},\eta_{-i}\right)\;\text{for}\;h\in c_{-i},\\ \alpha \int {\text{N}}_{d}\left(\eta_{i};\mu_{h},{\text{Δ}}_{h}\right)\text{dΠ}\left(\mu_{h},{\text{Δ}}_{h}\right)\;\text{for}\;h\notin c_{-i}. \end{array}$$

where $\eta_{-i}=\left\{\eta_{j}:j\neq i\right\}$ and $c_{-i}=\left\{c_{j}:j\neq i\right\}$. Due to conjugacy the above integrals are analytically available.

#### Step 5

Let r be the number of unique $c_{i}$’s. Following West (1992), first generate φ∼Beta(α+1,n), evaluate $\pi /(1-\pi )=\left(a_{\alpha }+r-1\right)/\left\{n\left(b_{\alpha }-\log\varphi \right)\right\}$ and generate

$$\alpha |\varphi ,r∼\{\begin{array}{l} \text{Ga}\left(\alpha +r,b_{\alpha }-\log\varphi \right)\;\text{with probability}\;\pi ,\\ \text{Ga}\left(\alpha +r-1,b_{\alpha }-\log\varphi \right)\;\text{with probability}\;1-\pi . \end{array}$$

#### Step 6

For j=1,…,p sample $\sigma_{j}^{2}$ from $\text{Ga}\;\left\{a_{\sigma }+n/2,b_{\sigma }+\sum_{n}^{i=1}{\left(y_{ij}-\lambda_{j}^{\text{T}}\eta_{i}\right)}^{2}/2\right\}$.

#### Step 7

Update the hyper-parameters of the Dirichlet-Laplace prior through:

- For j=1,…,p and h=1,…d sample ${\tilde{\psi }}_{jh}$ independently from an inverse-Gaussian $\text{iG}\left(\tau \phi_{jh}/\left|\lambda_{jh}\right|,1\right)$ distribution and set $\psi_{jh}=1/{\tilde{\psi }}_{jh}$.
- Sample the full conditional posterior distribution of τ from a generalized inverse Gaussian $\text{giG}\left\{dp(1-a),1,2\sum_{j,h}\left|\lambda_{jh}\right|/\phi_{jh}\right\}$ distribution.
- To sample ϕ∣Λ, draw $T_{jh}$ independently with $T_{jh}∼\text{giG}\left(a-1,1,2\left|\lambda_{jh}\right|\right)$ and set $\phi_{jh}=T_{jh}/T$ with $T=\sum_{jh}T_{jh}$.

This simple Gibbs sampler sometimes gets stuck in local modes; a key bottleneck is the exploration Step 4. Therefore, we adopt the split-merge MCMC procedure proposed by Jain and Neal (2004); the authors note that the Gibbs sampler is useful in moving singleton samples between clusters while the split-merge algorithm makes major changes. Hence, we randomly switch between Gibbs and split-merge updates. The split-merge algorithm makes smart proposals by performing restricted Gibbs scans of the same form as in (9).

From the posterior samples of $c_{i}$’s, we compute summaries following Wade and Ghahramani (2018). Our point estimate is the partition visited by the MCMC sampler that minimizes the posterior expectation of the Binder loss (Binder, 1978) exploiting the posterior similarity matrix obtained from the different sampled partitions.

The sampling algorithm can be easily modified for other priors on Λ having a conditionally Gaussian representation, with Step 7 modified accordingly. For example, we could use horseshoe (Carvalho et al., 2009), increasing shrinkage priors (Bhattacharya and Dunson, 2011; Legramanti et al., 2020; Schiavon et al., 2021), or the fast factor analysis prior (Ročková and George, 2016). Similarly, alternative priors for $\left\{\pi_{h}\right\}$, such as Pitman and Yor (1997) or Miller and Harrison (2018), can be adopted with minor modifications in Steps 4 and 5.

## 4. Properties of the Latent Mixture for Bayesian Clustering Method

### 4.1 Bayes Oracle Clustering Rule

We first define a Bayes oracle clustering rule where the observed data follow the distribution in model (5), that is, the high dimensional $y_{i}$’s provide error-prone measurements on unobserved lower-dimensional latent variables $\eta_{i}$’s on subject i, and we assume the oracle has knowledge of the exact values of the latent variables $\left\{\eta_{0i}\right\}$, where $\eta_{0i}$’s are d-dimensional latent vectors. Given this knowledge, the oracle can define any Bayesian mixture model to induce a posterior clustering of the data, which is not affected by the high-dimensionality of the problem. This leads to the distribution over the space of partitions in the following definition.

### Definition 4

*Let* $\eta_{0}=\left\{\eta_{01},\ldots ,\eta_{0n}\right\}$ *be the true values of the unobserved latent variables corresponding to each data point*. *The following mixture model is assumed to cluster* $\eta_{0}$

$$\eta_{0i}∼\sum_{h=1}^{\infty }\;\pi_{h}K\left(\eta_{0i};\theta_{h}\right),\;\left\{\pi_{h}\right\}∼Q_{0},\;\theta_{h}\overset{\text{iid}}{∼}G_{0}.$$

*Then the oracle probability of clustering is defined as*

(10) $$\text{Π}\left(\text{Ψ}|\eta_{0}\right)=\frac{\text{Π}(\text{Ψ})\times \int \prod_{h\geq 1}\prod_{i:c_{i}=h}K\left(\eta_{0i};\theta_{h}\right)\text{d}G_{0}\left(\theta_{h}\right)}{\sum_{{\text{Ψ}}^{\prime }\in P}\text{Π}\left({\text{Ψ}}^{\prime }\right)\times \int \prod_{h\geq 1}\prod_{i:c_{i}^{\prime }=h}K\left(\eta_{0i};\theta_{h}\right)\text{d}G_{0}\left(\theta_{h}\right)}.$$

Probability (10) expresses the oracles’ uncertainty in clustering if the clustering model could have been applied on the true latent factors. This is a gold standard in being free of the curse of dimensionality through using the oracles’ knowledge of the true latent variables, but we make no claims about the relationship between the oracle posterior and any ‘true’ clustering. Under the framework of Section 3, the high-dimensional measurements on each subject provide information on these latent variables, with the clustering done on the latent variable level. Ideally, we would get closer to the oracle partition probability under the proposed method as p increases, turning the curse of dimensionality into a blessing. We show that this is indeed the case in Section 4.3.

To this end, we assume the oracle uses a location mixture of Gaussians with a common covariance matrix. We assume the following mixture distribution on $\eta_{0i}$’s, independent non-informative Jeffreys prior for the common covariance and arbitrary prior $Q_{0}$ on the mixture probabilities:

(11) $$\eta_{i}\overset{\text{iid}}{∼}\sum_{h=1}^{\infty }\;\pi_{h}{\text{N}}_{d}\left(\mu_{h},\text{Δ}\right),{\;\mu_{h}\left|\text{Δ}\overset{\text{iid}}{∼}{\text{N}}_{d}\left(0,\kappa_{0}^{-1}\text{Δ}\right),\;\text{Δ}\propto \right|\text{Δ}|}^{-\frac{d+1}{2}},\;\left\{\pi_{h}\right\}∼Q_{0}.$$

For d<n, the oracle rule is well defined for the Jeffreys prior on Δ. Note that the marginal Jeffrey’s prior is free of any hyperparameter.

### 4.2 Assumptions on Data and Prior Specifications

In this section, we show that the posterior probability on the space of partitions induced by the proposed model converges to the oracle probability p→∞ as in expectation under appropriate conditions on the data generating process and the prior. We assume that the residual error variances $\sigma_{i}^{2}$’s are the same having true common value $\sigma_{0}^{2}$ for all j=1,…,p. Our result is based on the following assumptions on ${\mathbb{P}}_{0}^{p}$, the true data-generating distribution of $y_{1},\ldots ,y_{n}$:

(C1) $y_{i}\overset{\text{ind}}{∼}{\text{N}}_{p}\left({\text{Λ}}_{0}\eta_{0i},\sigma_{0}^{2}I_{p}\right)$, for each i=1,…,n;
(C2) $\lim_{p\to \infty }\;{\left\|\frac{1}{p}{\text{Λ}}_{0}^{\text{T}}{\text{Λ}}_{0}-M\right\|}_{2}=0$ where M is a d×d positive-definite matrix;
(C3) $\sigma_{L}^{2}<\sigma_{0}^{2}<\sigma_{U}^{2}$ where $\sigma_{L}^{2}$ and $\sigma_{U}^{2}$ are known constants;
(C4) $\left\|\eta_{0i}\right\|=O(1)$ for each i=1,…,n.

Condition (C1) corresponds to the conditional likelihood of $y_{i}$ given $\eta_{i}$ being correctly specified and the data containing increasing information on the latent factors as p increases. This increasing information assumption is extremely mild; indeed, each individual $y_{ij}$ can be very noisy and provide minimal information about $\eta_{i}$ and there will still be a build up of information across j=1,…,p as long as the additional variables are not completely uncorrelated with the target latent factors. In fact, we have a build up of information even when a proportion of the factor loadings are exactly zero, the factor loadings are very small relative to the residual variance, and the residuals are heavy-tailed. We illustrate this empirically with a simple simulation study in Section S.4.1 of the supplementary materials. Condition (C2) ensures that ${\text{Λ}}_{0}$ is not *ill-conditioned* and its spectral norm does not increase too fast with respect to p since the highest and lowest eigenvalues of ${\text{Λ}}_{0}^{\text{T}}{\text{Λ}}_{0}$ grow in O(p). Related but much stronger conditions appear in the factor modeling (Fan et al., 2008, 2011) and massive covariance estimation literature (Pati et al., 2014). We allow the columns of ${\text{Λ}}_{0}$ to be non-orthogonal with varying average squared values which is expected in high-dimensional studies. Condition (C3) bounds the variance of the observed $y_{i}\text{s}$ and (C4) is a weak assumption ensuring that the latent variables do not depend on n or p. Additionally, we assume that the latent dimension d is known.

Although we use a stick-breaking prior on the mixture probabilities $\left\{\pi_{h}\right\}$ in Section 3.1, we derive our results for an arbitrary prior $Q_{0}$ for wider applicability. We assume the inverse-gamma prior on residual variance $\sigma^{2}$ to be restricted to the compact set [$\sigma_{L}^{2}$, $\sigma_{U}^{2}$].

#### 4.3 Main Results

In Lemma 5 we derive sufficient conditions for the posterior probability on the space of partitions to converge to the oracle probability for p→∞.

#### Lemma 5

*Let* $\eta ={\left[\eta_{1},\ldots ,\eta_{n}\right]}^{\text{T}}$, $\zeta^{\left(p\right)}={\left[\zeta_{1}^{\left(p\right)},\ldots ,\zeta_{n}^{\left(p\right)}\right]}^{\text{T}}={\left(\sqrt{p\log p}\right)}^{-1}{\left({\text{Λ}}^{\text{T}}\text{Λ}\right)}^{1/2}\eta$ *and, for any* δ>0, $B_{p,\delta }=\overset{n}{\underset{i=1}{\cap }}\left\{\text{Λ},\eta_{i}:{\left(\sqrt{p\log p}\right)}^{-1}\|\text{Λ}\eta_{i}-{\text{Λ}}_{0}\eta_{0i}\|<\delta \right\}$. *Assume for any* δ>0

(12) $$\text{Π}\left({\bar{B}}_{p,\delta }|Y\right)\to 0\;{\mathbb{P}}_{0}^{p}\text{-a.s.}$$

*where* ${\bar{B}}_{p,\delta }$ *is the complement of* $B_{p,\delta }$. *Let* E(⋅∣Y) *denote expectation with respect to the posterior distribution of the parameters given data* Y *and* $\text{Π}\left(\text{Ψ}|\zeta^{\left(p\right)}\right)$ *be the conditional probability of partition* Ψ *with* $\eta_{0}$ *replaced by* $\zeta^{\left(p\right)}$ *in* (10). *Then*, $\lim_{p\to \infty }E\left\{\text{Π}\left(\text{Ψ}|\zeta^{\left(p\right)}\right)|Y\right\}=\text{Π}\left(\text{Ψ}|\eta_{0}\right)$.

In the following theorem, we show that condition (12) holds for Lamb and hence we avoid the large p pitfall. The proof is in the supplementary materials.

#### Theorem 6

*Let* $B_{p,\delta }$ *be as defined in Lemma 5 and* ${\bar{B}}_{p,\delta }$ *be its complement set*. *Then, under (C1)-(C4) and model* (6), $\text{Π}\left({\bar{B}}_{p,\delta }|Y\right)\to 0{\mathbb{P}}_{0}^{p}\text{-a.s}$. *for any* δ>0.

Theorem 6 implies that our model learns the latent factors more accurately with increasing p. In addition to the proof of Theorem 6, this result is further illustrated empirically via a simple simulation experiment reported in Section S.4.2 of the supplementary materials.

The oracle has a slightly simpler model specification than (8) assuming common covariances across components. This simplification is done to make the associated theory more tractable, but the simplified location mixture case is rich enough to provide a nice test case for assessing how the proposed approach can escape the curse of dimensionality.

As conditions (C1)-(C4) imply (A0), the clustering models in Corollaries 2 and 3 would still lead to the two extreme partitions. The Lamb model, in learning the low-dimensional latent space with increasing dimensions, escapes these pitfalls.

## 5. Simulation Study

We perform a simulation study to analyze the performance of Lamb in clustering high dimensional data. The sampler introduced in Section 3.2 is available from the GitHub page of the first author. We compare with a Dirichlet process mixture of Gaussian model with diagonal covariance matrix implemented in R package BNPmix (Corradin et al., 2021), a nonparametric mixture of infinite factor analyzers implemented in R package IMIFA (Murphy et al., 2019), and a pragmatic two-stage approach (PCA-KM) that performs an approximate sparse principal component analysis of the high dimensional data to reduce dimensionality from p to $\hat{d}$—with $\hat{d}$ the minimum number of components explaining at least 95% of the variability as discussed in Section 3.1—and then applies k-means on the principal components, with k chosen by maximizing the average silhouette width (Rousseeuw, 1987). This same approach is used to choose $\hat{d}$ in implementing Lamb.

For the high-dimensional simulation settings we considered, both the mixture of Gaussians and the mixture of factor analyzers showed high instability, including software crashing for memory issues, lack of convergence, and extremely long running times. For these reasons we report a comparison with PCA-KM approach only. To test the accuracy of the estimated clustering relative to the true clustering, we compute the adjusted Rand index (Rand, 1971).

We generated data under: [1] Lamb, [2] mixture of sparse factor analyzers (MFA), and [3] mixture of log transformed zero inflated sparse Poisson counts (SpCount) [1]-[2] have latent dimension 20, while for [3] the data are discrete and highly non-Gaussian within clusters mimicking the data of Section 6. Details are provided in Section S.3 of the supplementary materials.

We vary true number of clusters $k_{0}\in \{10,15,25\}$, with the first $\left\lfloor 2k_{0}/3\right\rfloor$ ‘main’ clusters having the same probability and the remaining ones having together the same probability of a single main cluster. For example if $k_{0}=25$, we set 16 main clusters with probability 1*/*17 each and 9 minor clusters of equal weights, whose total probability sums to 1*/*17. This is a highly challenging case, as many methods struggle unless there are a small number of close to equal weight clusters that are well separated. The dimension p varies in p={1,000,2,500} while the sample size n is n=2,000. Data visualization plots using McInnes et al. (2018) are in Section S.4.4 of the supplementary materials. For each configuration, we perform 20 independent replications. We run our sampler for 6,000 iterations discarding the first 1,000 as burn in and taking one draw every five to reduce autocorrelation. Prior elicitation follows the default specification of Section 3.1. On average, 6,000 iterations under these settings took between 40 and 50 minutes on a iMac with 4.2 GHz Quad-Core Intel Core i7 processor and 32GB DDR4 RAM.

**Figure 1:**
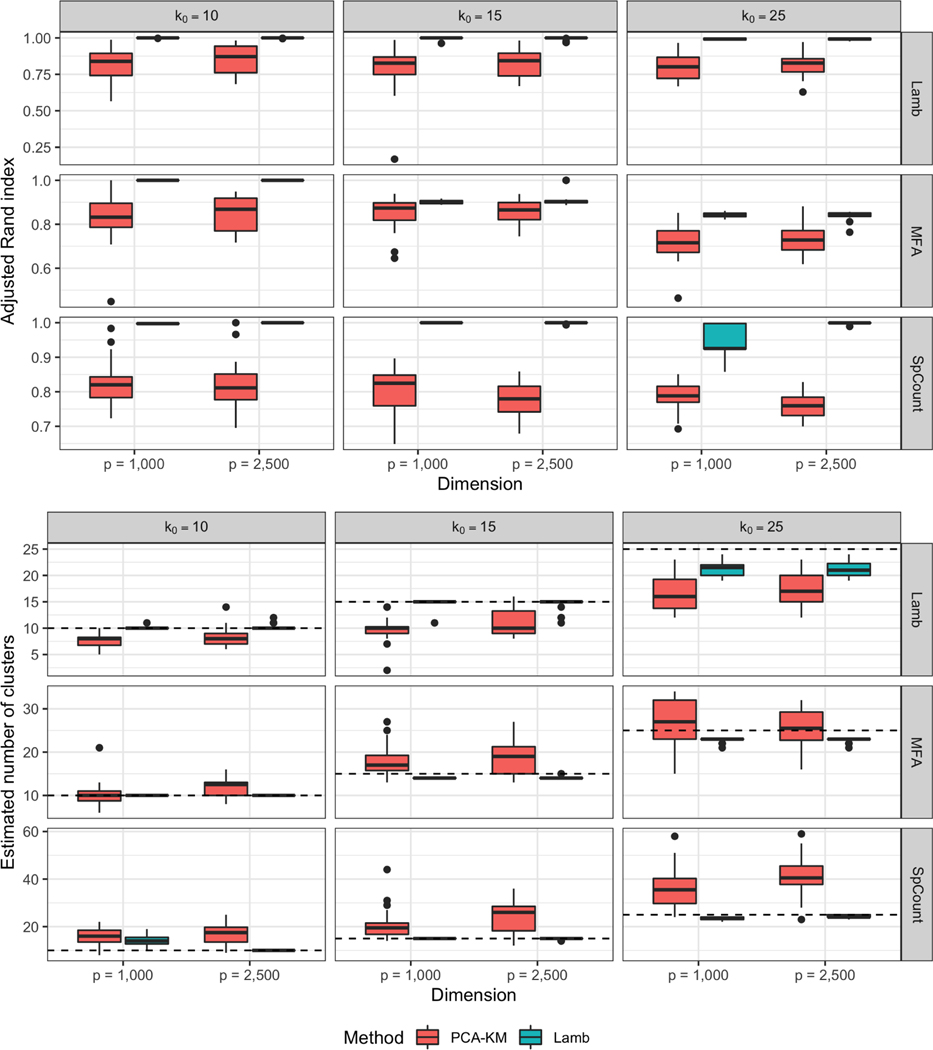
Comparison between our proposed Lamb and the two-stage PCA-KM approach: Distributions of the adjusted Rand indices (upper plot) and estimated number of clusters (lower plot) in 20 replicated experiments. Horizontal dashed lines denote the true number of clusters. The simulation scenarios, reported in each row, are labeled as Lamb for the model of Section 3, MFA for mixture of factor analyzers and SpCount for the log transformed zero inflated sparse Poisson counts.

Figure 1 reports the distribution of the 20 replicates of the adjusted Rand index and mean estimated number of clusters. Our proposed Lamb is uniformly superior in each scenario obtaining high adjusted Rand indices, accurate clustering results, and less variability across replicates. In the MFA scenario, Lamb yields relatively lower Rand index for $k_{0}=25$. This is not unusual due to model misspecification and the large number of clusters.

The Lamb results do not vary much across the simulation replicates because the oracle posterior is quite concentrated at the true clustering. Since the dimension p is in the thousands, the asymptotic results derived in Section 4 kicked in resulting in narrow posterior credible intervals. To understand the performance of our proposed method in smaller sample sizes, we include additional simulation results with n=500 in Section S.4.3 of the supplementary materials.

Furthermore, Section S.4.1 in the supplementary materials reports two simple simulation experiments showing that the degenerate clustering behavior discussed in Section 2 is evident even in moderate dimensions of p=20.

## 6. Application to ScRNASeq Cell Line Dataset

In this section, we analyze the GSE81861 cell line dataset (Li et al., 2017) to illustrate the proposed method. The dataset profiles 630 cells from 7 cell lines using the Fluidigm based single cell RNA-seq protocol (See et al., 2018). The dataset includes 83 A549 cells, 65 H1437 cells, 55 HCT116 cells, 23 IMR90 cells, 96 K562 cells, 134 GM12878 cells, 174 H1 cells and 57,241 genes. The cell types are known and hence the data provide a useful benchmark to assess performance in clustering high-dimensional data.

Following standard practice in single cell data analysis, we apply data pre-processing. Cells with low read counts are discarded, as we lack reliable gene expression measurements for these cells, and data are normalized following Lun et al. (2016). We remove non-informative genes using M3Drop (Andrews and Hemberg, 2018). After this pre-processing phase, we obtain a final dataset with n=531 cells and p=7,666 genes.

Applying our empirical Bayes approach, we estimate the latent dimension as $\hat{d}=19$. We implement Lamb using our default prior, collecting 10, 000 iterations after a burn-in of 5, 000 and keeping one draw in five. MCMC converge diagnostics are provided in Section S.5 in the supplementary materials. As comparison, we apply the two stage procedure of the previous section and the popular Seurat (Butler et al., 2018) pipeline which performs quality control, normalization, and selects informative genes that exhibit high variation across the cells.

**Figure 2:**
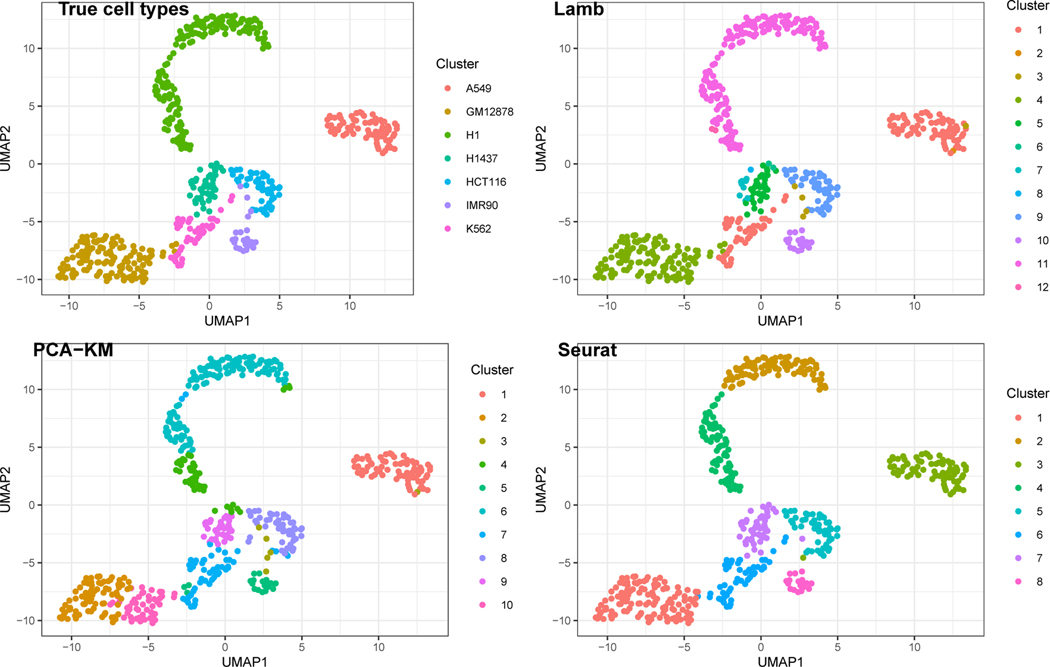
UMAP plots of the cell line dataset: Clusterings corresponding to the true cell-types, Lamb estimate, PCA-KM estimate and Seurat estimate are plotted in clockwise manner. Different panels use different color legends.

Graphical representations of the different clustering results are shown in Figure 2 via UMAP projections (McInnes et al., 2018). Our proposed Lamb, the two stage approach, and Seurat achieve adjusted Rand indices of 0.977, 0.734 and 0.805 when compared to the true cluster-configuration and yield 12, 10, and 8 clusters, respectively. Seurat is reasonably accurate but splits the H1 cell-type into two clusters, while the two-stage approach is dramatically worse.

**Figure 3:**
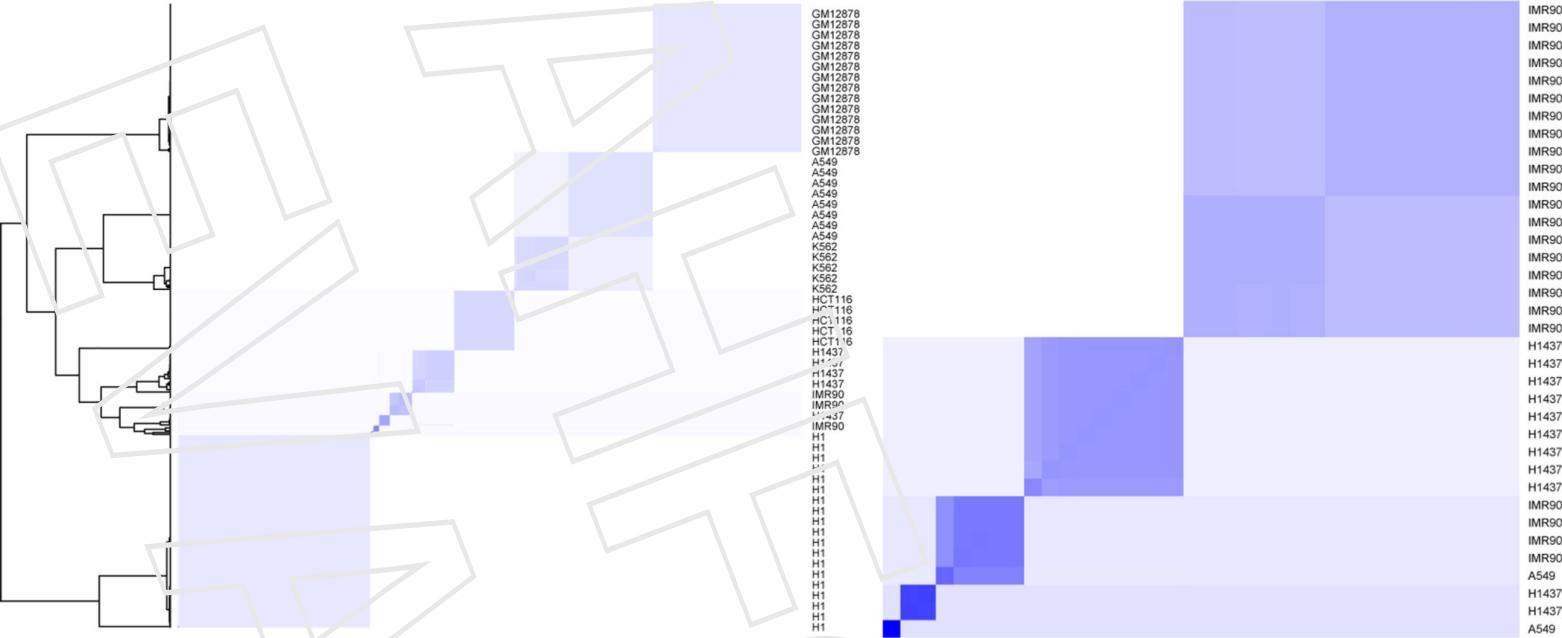
Posterior similarity matrix obtained from the Markov chain Monte Carlo samples of the Lamb method: Left panel reports the similarity matrix for the full cell line dataset along with the dendrogram obtained using complete linkage; row names report the true cluster names; right panel zooms the center of the left panel.

An appealing aspect of our approach is posterior uncertainty quantification. The 95% credible interval for the adjusted Rand index is [0.900, 0.985] and the posterior probability of having between 11 and 13 clusters is 0.98. This suggests that the posterior distribution is highly concentrated, which is consistent with our simulations. The posterior similarity matrix reported in the first panel of Figure 3—also reporting the related dendrogram obtained by using complete linkage—clearly shows that the majority of the observations have a high posterior probability of being assigned to a specific cluster and negligible probability of being assigned to artifactual clusters. Figure 3 also shows micro clusters leading to over-estimation of the number of cell types. Two cells of cluster A549 are put in singleton clusters. Similarly cluster IMR90 is divided into two clusters of size 4 and 19 with negligible posterior probability of being merged. Finally cluster H1437 is split into four clusters with the main one comprising 35 of 47 observations and the smallest one comprising just one observation. Such micro-clusters have negligible impact for practical inference since Lamb does recover the original clustering configurations for most cell-types as reflected by the high adjusted Rand index with the true cell-types. Single-cell experiments are subject to high technical noise (Brennecke et al., 2013) which is not possible to completely remove in pre-processing steps. Such noise can potentially induce differences between cells that may not have any biological significance, for example, the cells in IMR90 (split into the clusters 3 and 10, see the top panel of Figure 2 for details) exhibit a substantial amount of variability although they are biologically of the same type.

## 7. Discussion

Part of the appeal of Bayesian methods is the intrinsic penalty for model complexity or ‘Bayesian Ockham razor’ (Jefferys and Berger, 1992), which comes through integrating the likelihood over the prior in obtaining the marginal likelihood. If one adds unnecessary parameters, then the likelihood is integrated over a larger region, which tends to reduce the marginal likelihood. In clustering problems, one relies on the Bayesian Ockham razor to choose the appropriate compromise between the two extremes of too many clusters and over-fitting and too few clusters and under-fitting. Often in low-dimensional problems, this razor is effective and one obtains a posterior providing a reasonable representation of uncertainty in clustering data into groups of relatively similar observations. However, a key contribution of this article is showing that this is fundamentally not the case in high-dimensional problems, and one can obtain nonsensical results using seemingly reasonable priors.

Perhaps our most interesting result is the degenerate behavior in the p→∞ case for the true posterior on clusterings, regardless of the true data generating model. This negative result provided motivation for our latent factor mixture model, which addresses the large p pitfall by clustering on the latent variable level. Using a low rank factorization with appropriate shrinkage priors, the method can also handle realistic high-dimensional problems. Another interesting theoretical result is our notion of a Bayesian oracle for clustering; to our knowledge, there is not a similar concept in the literature. We show that our proposed Lamb attains the oracle with increasing dimensions.

Several interesting projects stem from the proposed work, which is a first step towards addressing pitfalls of Bayesian approaches to high-dimensional clustering. One important thread is designing faster MCMC algorithms for massive sample size exploiting parallel and distributed computing; for example, running MCMC for different subsets of the variables in parallel and combining the results. Some recent works in the literature discuss related approaches (Ni et al., 2020; Song et al., 2020) but without considering the pitfalls that arise in high-dimensional data clustering. Another thread is to develop fast approximate inference algorithms that avoid MCMC, such as variational Bayes. In addition, it is of substantial interest to generalize the proposed approach to handle more complex data structures; for example, involving data that are not real-valued vectors and allowing for kernel misspecification (Miller and Dunson, 2019). In our settings d and n are fixed and not growing with p. The study of situations in which p, d and n jointly increase—at some rate—would be a very interesting theoretical extension of our results.

## Supplementary Material

1

## Appendix

### Proofs of Section 2

**Proof** [Theorem 1] Consider the ratio of posterior probabilities:

(A.1)
$$\frac{\text{Π}(\text{Ψ}|Y)}{\text{Π}\left({\text{Ψ}}^{\prime }|Y\right)}.$$

If this ratio converges to zero for all $c_{1},\ldots ,c_{n}$ in ${\mathbb{P}}_{0}^{p}$-probability as p→∞, then any partition nested into another partition is more likely *a posteriori* implying $\text{Π}\left(c_{1}=\cdots =c_{n}|Y\right)=1$ in ${\mathbb{P}}_{0}^{p}$-probability so that all subjects are grouped in the same cluster with probability one. Conversely if the ratio converges to +∞, then $\text{Π}\left(c_{1}\neq \cdots \neq c_{n}|Y\right)=1$ in ${\mathbb{P}}_{0}^{p}$-probability and each subject is assigned to their own cluster with probability one.

Without loss of generality, assume that $c_{1},\ldots ,c_{n}$ define $k_{n}$ clusters of sizes $n_{1},\ldots ,n_{k_{n}}$ and that $c_{i}^{\prime }=c_{i}$ for $c_{i}\in \left\{1,\ldots ,k_{n}-2\right\}$ and $c_{i}^{\prime }=k_{n}-1$ for $c_{i}\in \left\{k_{n}-1,k_{n}\right\}$, with $n_{1}^{\prime },\ldots ,n_{k_{n}^{\prime }-1}^{\prime }$ the cluster sizes under the partition induced by the $c_{i}^{\prime }$. In general, ratio (A.1) can be expressed as

(A.2)
$$\frac{\text{Π}(\text{Ψ})}{\text{Π}\left({\text{Ψ}}^{\prime }\right)}\times \frac{\prod_{h=1}^{k_{n}}\;\int \prod_{i:c_{i}=h}K\left(y_{i};\theta \right)\text{d}P_{0}(\theta )}{\prod_{h=1}^{k_{n}-1}\;\int \prod_{i:c_{i}^{\prime }=h}K\left(y_{i};\theta \right)\text{d}P_{0}(\theta )}.$$

The left hand side of (A.2) can be expressed as the ratio between the EPPFs. Since by assumption there is a positive prior probability for any partition in P, this ratio is finite and does not depend on p or the data generating distribution. Thus, by induction and under the assumptions on the right factor of (A.2) we conclude the proof. ■

**Proof** [Corollary 2] Define $c_{1},\ldots ,c_{n}$ and $c_{1}^{\prime },\ldots ,c_{n}^{\prime }$ consistently with the proof of Theorem 1. Then, consider the ratio of the marginal likelihoods

(A.3)
$$\frac{\prod_{h=1}^{k_{n}}\int \prod_{i:c_{i}=h}{\text{N}}_{p}\left(y_{i};\mu_{h},{\text{Σ}}_{h}\right){\text{N}}_{p}\left(\mu_{h};\mu_{0},\kappa_{0}^{-1}{\text{Σ}}_{h}\right)IW\left({\text{Σ}}_{h};\nu_{0},{\text{Λ}}_{0}\right)\text{d}\left(\mu_{h},{\text{Σ}}_{h}\right)}{\prod_{h=1}^{k_{n}-1}\;\int \prod_{i:c_{i}^{\prime }=h}{\text{N}}_{p}\left(y_{i};\mu_{h},{\text{Σ}}_{h}\right){\text{N}}_{p}\left(\mu_{h};\mu_{0},\kappa_{0}^{-1}{\text{Σ}}_{h}\right)IW\left({\text{Σ}}_{h};\nu_{0},{\text{Λ}}_{0}\right)\text{d}\left(\mu_{h},{\text{Σ}}_{h}\right)}.$$

The numerator of (A.3) is

$$\prod_{h=1}^{k_{n}}\;\left\{\frac{1}{\pi^{n_{h}p/2}}\frac{{\text{Γ}}_{p}\left(\frac{\nu_{0}+n_{h}}{2}\right)}{{\text{Γ}}_{p}\left(\frac{\nu_{0}}{2}\right)}{\left(\frac{\kappa_{0}}{\kappa_{0}+n_{h}}\right)}^{\frac{p}{2}}\frac{{\left|{\text{Λ}}_{0}\right|}^{\frac{\nu_{0}}{2}}}{{\left|{\text{Λ}}_{0}+S_{h}^{\text{Ψ}}+\frac{n_{h}\kappa_{0}}{\kappa_{0}+n_{h}}\left({\bar{y}}_{h}^{\text{Ψ}}-\mu_{0}\right){\left({\bar{y}}_{h}^{\text{Ψ}}-\mu_{0}\right)}^{\text{T}}\right|}^{\frac{\nu_{0}+n_{h}}{2}}}\right\},$$

with ${\bar{y}}_{h}^{\text{Ψ}}=n_{h}^{-1}\sum_{i:c_{i}=h}y_{i}$, $S_{h}^{\text{Ψ}}=\sum_{i:c_{i}=h}\left(y_{i}-{\bar{y}}_{h}^{\text{Ψ}}\right){\left(y_{i}-{\bar{y}}_{h}^{\text{Ψ}}\right)}^{\text{T}}$, and ${\text{Γ}}_{p}(\cdot )$ being the multivariate gamma function. Obtaining a corresponding expression for the denominator, the ratio (A.3) becomes

(A.4)
$$\frac{{\text{Γ}}_{p}\left(\frac{\nu_{0}+n_{k_{n}-1}}{2}\right){\text{Γ}}_{p}\left(\frac{\nu_{0}+n_{k_{n}}}{2}\right)}{{\text{Γ}}_{p}\left(\frac{\nu_{0}+n_{k_{n}-1}^{\prime }}{2}\right){\text{Γ}}_{p}\left(\frac{\nu_{0}}{2}\right)}\times {\left\{\frac{\kappa_{0}\left(\kappa_{0}+n_{k_{n}-1}^{\prime }\right)}{\left(\kappa_{0}+n_{k_{n}-1}\right)\left(\kappa_{0}+n_{k_{n}}\right)}\right\}}^{p/2}\;\;\times \frac{{\left|{\text{Λ}}_{0}\right|}^{\frac{\nu_{0}}{2}}{\left|{\text{Λ}}_{0}+S_{k_{n}-1}^{{\text{Ψ}}^{\prime }}+\frac{n_{k_{n}-1}^{\prime }\kappa_{0}}{\kappa_{0}+n_{k_{n}-1}^{\prime }}\left({\bar{y}}_{k_{n}-1}^{{\text{Ψ}}^{\prime }}-\mu_{0}\right){\left({\bar{y}}_{k_{n}-1}^{{\text{Ψ}}^{\prime }}-\mu_{0}\right)}^{\text{T}}\right|}^{\frac{\nu_{0}+n_{k_{n}-1}^{\prime }}{2}}}{\prod_{h=k_{n}-1}^{k_{n}}\;{\left|{\text{Λ}}_{0}+S_{h}^{\text{Ψ}}+\frac{n_{h}\kappa_{0}}{\kappa_{0}+n_{h}}\left({\bar{y}}_{h}^{\text{Ψ}}-\mu_{0}\right){\left({\bar{y}}_{h}^{\text{Ψ}}-\mu_{0}\right)}^{\text{T}}\right|}^{\frac{\nu_{0}+n_{h}}{2}}}.$$

We first study the limit of the first factor of (A.4). From Lemma S.7, we have

$$\underset{p\to \infty }{\lim}\;\frac{1}{p}\left\{\log\frac{{\text{Γ}}_{p}\left(\frac{\nu_{0}+n_{k_{n}}}{2}\right)}{{\text{Γ}}_{p}\left(\frac{\nu_{0}+n_{k_{n}-1}^{\prime }}{2}\right)}+\log\frac{{\text{Γ}}_{p}\left(\frac{\nu_{0}+n_{k_{n}-1}}{2}\right)}{{\text{Γ}}_{p}\left(\frac{\nu_{0}}{2}\right)}\right\}=0.$$

We now study the limit of the remaining part of (A.4). Note that, if we replace each observation $y_{i}$ with ${\tilde{y}}_{i}={\text{Λ}}_{0}^{-1/2}\left(y_{i}-\mu_{0}\right)$, assumption (A0) is still valid for ${\tilde{y}}_{i}$’s. Moreover, $\left|{\text{Λ}}_{0}\right|$ terms get canceled out from (A.4). Hence, without loss of generality we can assume $\mu_{0}=0$ and ${\text{Λ}}_{0}=I_{p}$. Without loss of generality we can also assume that $y_{1+\sum_{j=1}^{h-1}\;n_{j}},\ldots ,y_{\sum_{j=1}^{h}\;n_{j}}$ are in cluster h. We define

$$Y_{\left(h\right)}^{\text{Ψ}}={\left[y_{1+\sum_{j=1}^{h-1}\;n_{j}},\ldots ,y_{\sum_{j=1}^{h}\;n_{j}}\right]}^{\text{T}},$$

to be the sub-data matrix corresponding to the h-th cluster in partition Ψ. Exploiting lower rank factorization results on matrix determinants, we have

$$\;\left|I_{p}+S_{h}^{\text{Ψ}}+\frac{n_{h}\kappa_{0}}{n_{h}+\kappa_{0}}{\bar{y}}_{h}^{\text{Ψ}}{\bar{y}}_{h}^{{\text{Ψ}}^{\text{T}}}\right|=\left|I_{p}+Y_{\left(h\right)}^{{\text{Ψ}}^{\text{T}}}Y_{\left(h\right)}^{\text{Ψ}}-\frac{1}{n_{h}+\kappa_{0}}Y_{\left(h\right)}^{{\text{Ψ}}^{\text{T}}}1_{n_{h}}1_{n_{h}}^{\text{T}}Y_{\left(h\right)}^{\text{Ψ}}\right|\;=\left|1-\frac{1}{n_{h}+\kappa_{0}}1_{n_{h}}^{\text{T}}Y_{\left(h\right)}^{\text{Ψ}}{\left\{I_{p}+Y_{\left(h\right)}^{{\text{Ψ}}^{\text{T}}}Y_{\left(h\right)}^{\text{Ψ}}\right\}}^{-1}Y_{\left(h\right)}^{{\text{Ψ}}^{\text{T}}}1_{n_{h}}\right||I_{p}+Y_{\left(h\right)}^{{\text{Ψ}}^{\text{T}}}Y_{\left(h\right)}^{\text{Ψ}}|,$$

where the symbol |A| or |a| is to be interpreted as the determinant of the matrix A or the absolute value of the scalar a, respectively. Then, the second factor of (A.4) simplifies to

$$\frac{{\left\{\left|1-\frac{1}{n_{k_{n}-1}^{\prime }+\kappa_{0}}1_{n_{k_{n}-1}^{\prime }}^{\text{T}}Y_{\left(k_{n}-1\right)}^{{\text{Ψ}}^{\prime }}{\left(I_{p}+Y_{\left(k_{n}-1\right)}^{{\text{Ψ}}^{\prime \text{T}}}Y_{\left(k_{n}-1\right)}^{{\text{Ψ}}^{\prime }}\right)}^{-1}Y_{\left(k_{n}-1\right)}^{{\text{Ψ}}^{\prime \text{T}}}1_{n_{k_{n}-1}^{\prime }}\right|\left|I_{p}+Y_{\left(k_{n}-1\right)}^{{\text{Ψ}}^{\prime \text{T}}}Y_{\left(k_{n}-1\right)}^{{\text{Ψ}}^{\prime }}\right|\right\}}^{\frac{\nu_{0}+n_{k_{n}-1}^{\prime }}{2}}}{\prod_{h=k_{n}-1}^{k_{n}}\;{\left\{\left|1-\frac{1}{n_{h}+\kappa_{0}}1_{n_{h}}^{\text{T}}Y_{\left(h\right)}^{\text{Ψ}}{\left(I_{p}+Y_{\left(h\right)}^{{\text{Ψ}}^{\text{T}}}Y_{\left(h\right)}^{\text{Ψ}}\right)}^{-1}Y_{\left(h\right)}^{{\text{Ψ}}^{\text{T}}}1_{n_{h}}\right|\left|I_{p}+Y_{\left(h\right)}^{{\text{Ψ}}^{\text{T}}}Y_{\left(h\right)}^{\text{Ψ}}\right|\right\}}^{\frac{\nu_{0}+n_{h}}{2}}}.$$

Using Lemma S.9, $\lim_{p\to \infty }\;{\left\|Y_{\left(h\right)}{\left\{I_{p}+Y_{\left(h\right)}^{\text{T}}Y_{\left(h\right)}\right\}}^{-1}Y_{\left(h\right)}^{\text{T}}-I_{n_{h}}\right\|}_{2}=0$ in ${\mathbb{P}}_{0}^{p}$-probability and

(A.5)
$$\underset{p\to \infty }{\lim}\;\left|1-\frac{1}{n_{h}+\kappa_{0}}1_{n_{h}}^{\text{T}}Y_{\left(h\right)}{\left\{I_{p}+Y_{\left(h\right)}^{\text{T}}Y_{\left(h\right)}\right\}}^{-1}Y_{\left(h\right)}^{\text{T}}1_{n_{h}}\right|=\frac{\kappa_{0}}{\kappa_{0}+n_{h}}\;\text{in}\;{\mathbb{P}}_{0}^{p}\text{-probability}.$$

Taking the logarithm of the second and third factor of (A.4) and rearranging it using the previous result

(A.6)
$$\log\frac{{\left|1-\frac{1}{n_{k_{n}-1}^{\prime }+\kappa_{0}}1_{n_{k_{n}-1}^{\prime }}^{\text{T}}Y_{\left(k_{n}-1\right)}^{{\text{Ψ}}^{\prime }}{\left\{I_{p}+Y_{\left(k_{n}-1\right)}^{{\text{Ψ}}^{\prime \text{T}}}Y_{\left(k_{n}-1\right)}^{{\text{Ψ}}^{\prime }}\right\}}^{-1}Y_{\left(k_{n}-1\right)}^{{\text{Ψ}}^{\prime \text{T}}}1_{n_{k_{n}-1}^{\prime }}\right|}^{\frac{\nu_{0}+n_{k_{n}-1}^{\prime }}{2}}}{\prod_{h=k_{n}-1}^{k_{n}}\;{\left|1-\frac{1}{n_{h}+\kappa_{0}}1_{n_{h}}^{\text{T}}Y_{\left(h\right)}^{\text{Ψ}}{\left\{I_{p}+Y_{\left(h\right)}^{{\text{Ψ}}^{\text{T}}}Y_{\left(h\right)}^{\text{Ψ}}\right\}}^{-1}Y_{\left(h\right)}^{{\text{Ψ}}^{\text{T}}}1_{n_{h}}\right|}^{\frac{\nu_{0}+n_{h}}{2}}}\;\;+\log{\left\{\frac{\kappa_{0}\left(\kappa_{0}+n_{k_{n}-1}^{\prime }\right)}{\left(\kappa_{0}+n_{k_{n}-1}\right)\left(\kappa_{0}+n_{k_{n}}\right)}\right\}}^{p/2}+\log\frac{{\left|I_{p}+Y_{\left(k_{n}-1\right)}^{{\text{Ψ}}^{\prime T}}Y_{\left(k_{n}-1\right)}^{{\text{Ψ}}^{\prime }}\right|}^{\frac{\nu_{0}+n_{k_{n}-1}^{\prime }}{2}}}{\prod_{h=k_{n}-1}^{k_{n}}\;{\left|I_{p}+Y_{\left(h\right)}^{{\text{Ψ}}^{\text{T}}}Y_{\left(h\right)}^{\text{Ψ}}\right|}^{\frac{\nu_{0}+n_{h}}{2}}}.$$

Since $\nu_{0}=p+c$, in conjunction with (A.5) we have sum of the limits of the first and second terms in (A.6) is 0 in ${\mathbb{P}}_{0}^{p}$-probability. We finally study the last summand of (A.5) and particularly

(A.7)
$$\underset{p\to \infty }{\lim}\;\frac{1}{p}\log\frac{\left|I_{p}+Y_{\left(k_{n}-1\right)}^{{\text{Ψ}}^{\prime T}}Y_{\left(k_{n}-1\right)}^{{\text{Ψ}}^{\prime }}\right|\frac{\nu_{0}+n_{k_{n}-1}^{\prime }}{2}}{\prod_{h=k_{n}-1}^{k_{n}}\;{\left|I_{p}+Y_{\left(h\right)}^{{\text{Ψ}}^{\text{T}}}Y_{\left(h\right)}^{\text{Ψ}}\right|}^{\frac{\nu_{0}+n_{h}}{2}}}.$$

Since $\left|I_{p}+Y_{\left(h\right)}^{{\text{Ψ}}^{\text{T}}}Y_{\left(h\right)}^{\text{Ψ}}\right|=\left|I_{n_{h}}+Y_{\left(h\right)}^{\text{Ψ}}Y_{\left(h\right)}^{{\text{Ψ}}^{\text{T}}}\right|$ for any partition Ψ, following the same arguments of (S.3) from Lemma S.10 in the supplementary materials, we have

$$\left|I_{p}+Y_{\left(k_{n}-1\right)}^{{\text{Ψ}}^{\prime \text{T}}}Y_{\left(k_{n}-1\right)}^{{\text{Ψ}}^{\prime }}\right|=\prod_{h=k_{n}-1}^{k_{n}}\;{\left|I_{p}+Y_{\left(h\right)}^{{\text{Ψ}}^{\text{T}}}Y_{\left(h\right)}^{\text{Ψ}}\right|}^{\frac{\nu_{0}+n_{h}}{2}}\times \left|I_{n_{k_{n}-1}}-ZZ^{\text{T}}\right|$$

where $Z={\left\{I_{n_{k_{n}-1}}+Y_{\left(k_{n}-1\right)}^{\text{Ψ}}Y_{\left(k_{n}-1\right)}^{{\text{Ψ}}^{\text{T}}}\right\}}^{-1/2}Y_{\left(k_{n}-1\right)}^{\text{Ψ}}Y_{\left(k_{n}\right)}^{{\text{Ψ}}^{\text{T}}}{\left\{I_{n_{k_{n}}}+Y_{\left(k_{n}\right)}^{\text{Ψ}}Y_{\left(k_{n}\right)}^{{\text{Ψ}}^{\text{T}}}\right\}}^{-1/2}$, and (A.7) reduces to

$$\frac{n_{k_{n}}}{2p}\log\left|I_{n_{k_{n}-1}}+Y_{\left(k_{n}-1\right)}^{\text{Ψ}}Y_{\left(k_{n}-1\right)}^{{\text{Ψ}}^{\text{T}}}\right|+\frac{n_{k_{n}-1}}{2p}\log\left|I_{n_{k_{n}}}+Y_{\left(k_{n}\right)}^{\text{Ψ}}Y_{\left(k_{n}\right)}^{{\text{Ψ}}^{\text{T}}}\right|+\frac{\nu_{0}+n_{k_{n}-1}^{\prime }}{2p}\log\left|I_{n_{k_{n}-1}}-ZZ^{\text{T}}\right|.$$

From (A0), it can be deduced that the limits of the first two terms in the last expression are 0 in ${\mathbb{P}}_{0}^{p}$-probability as p→∞. Invoking Lemma S.10 and the fact that $\nu_{0}=p+c$, we have

$$\underset{p\to \infty }{\text{lim}\;\text{sup}}\frac{\nu_{0}+n_{k_{n}-1}^{\prime }}{2p}\log\left|I_{n_{k_{n}-1}}-ZZ^{\text{T}}\right|<0,$$

and henceforth (A.7) is negative. This leads to

$$\underset{p\to \infty }{\text{lim}\;\text{sup}}\log\frac{\prod_{h=1}^{k_{n}}\;\int \prod_{i:c_{i}=h}{\text{N}}_{p}\left(y_{i};\mu_{h},{\text{Σ}}_{h}\right){\text{N}}_{p}\left(\mu_{h};\mu_{0},\kappa_{0}^{-1}{\text{Σ}}_{h}\right)IW\left({\text{Σ}}_{h};\nu_{0},{\text{Λ}}_{0}\right)\text{d}\left(\mu_{h},{\text{Σ}}_{h}\right)}{\prod_{h=1}^{k_{n}-1}\;\int \prod_{i:c_{i}^{\prime }=h}{\text{N}}_{p}\left(y_{i};\mu_{h},{\text{Σ}}_{h}\right){\text{N}}_{p}\left(\mu_{h};\mu_{0},\kappa_{0}^{-1}{\text{Σ}}_{h}\right)IW\left({\text{Σ}}_{h};\nu_{0},{\text{Λ}}_{0}\right)\text{d}\left(\mu_{h},{\text{Σ}}_{h}\right)}=0,$$

and hence for p→∞ all the data points are cluster together in ${\mathbb{P}}_{0}^{p}$-probability thanks to Theorem 1. ■

**Proof** [Corollary 3] Define $c_{1},\ldots ,c_{n}$ and $c_{1}^{\prime },\ldots ,c_{n}^{\prime }$ consistently with the proof of Theorem 1. Then, consider the ratio of the marginal likelihoods

(A.8)
$$\frac{\int \prod_{h=1}^{k_{n}}\;\int \prod_{i:c_{i}=h}{\text{N}}_{p}\left(y_{i};\mu_{h},\text{Σ}\right){\text{N}}_{p}\left(\mu_{h};\mu_{0},\kappa_{0}^{-1}\text{Σ}\right)\text{d}\mu_{h}IW\left(\text{Σ};\nu_{0},{\text{Λ}}_{0}\right)\text{dΣ}}{\int \prod_{h=1}^{k_{n}-1}\;\int \prod_{i:c_{i}^{\prime }=h}{\text{N}}_{p}\left(y_{i};\mu_{h},\text{Σ}\right){\text{N}}_{p}\left(\mu_{h};\mu_{0},\kappa_{0}^{-1}\text{Σ}\right)\text{d}\mu_{h}IW\left(\text{Σ};\nu_{0},{\text{Λ}}_{0}\right)\text{dΣ}}.$$

The numerator of (A.8) is

$$\prod_{h=1}^{k_{n}}\;{\left(\frac{\kappa_{0}}{n_{h}+\kappa_{0}}\right)}^{\frac{p}{2}}{\left|{\text{Λ}}_{0}+\sum_{h=1}^{k_{n}}\;\left\{S_{h}^{\text{Ψ}}+\frac{n_{h}\kappa_{0}}{n_{h}+\kappa_{0}}\left({\bar{y}}_{h}^{\text{Ψ}}-\mu_{0}\right){\left({\bar{y}}_{h}^{\text{Ψ}}-\mu_{0}\right)}^{\text{T}}\right\}\right|}^{-\frac{\nu_{0}+n}{2}}\pi^{-\frac{np}{2}}\frac{{\text{Γ}}_{p}\left(\frac{\nu_{0}+n}{2}\right)}{{\text{Γ}}_{p}\left(\nu_{0}/2\right)}{\left|{\text{Λ}}_{0}\right|}^{\frac{\nu_{0}}{2}},$$

where ${\bar{y}}_{h}^{\text{Ψ}}=\frac{1}{n_{h}}\sum_{i:c_{i}=h}y_{i}$ and $S_{h}^{\text{Ψ}}=\sum_{i:c_{i}=h}\left(y_{i}-{\bar{y}}_{h}^{\text{Ψ}}\right){\left(y_{i}-{\bar{y}}_{h}^{\text{Ψ}}\right)}^{\text{T}}$. Hence, obtaining a corresponding expression for the denominator, ratio (A.8) becomes

(A.9)
$${\left\{\frac{\kappa_{0}\left(\kappa_{0}+n_{k_{n}-1}^{\prime }\right)}{\left(\kappa_{0}+n_{k_{n}-1}\right)\left(\kappa_{0}+n_{k_{n}}\right)}\right\}}^{\frac{p}{2}}{\left[\frac{\left|{\text{Λ}}_{0}+\sum_{h=1}^{k_{n}-1}\;\left\{S_{h}^{{\text{Ψ}}^{\prime }}+\frac{n_{h}^{\prime }\kappa_{0}}{n_{h}^{\prime }+\kappa_{0}}\left({\bar{y}}_{h}^{{\text{Ψ}}^{\prime }}-\mu_{0}\right){\left({\bar{y}}_{h}^{{\text{Ψ}}^{\prime }}-\mu_{0}\right)}^{\text{T}}\right\}\right|}{\left|{\text{Λ}}_{0}+\sum_{h=1}^{k_{n}}\;\left\{S_{h}^{\text{Ψ}}+\frac{n_{h}\kappa_{0}}{n_{h}+\kappa_{0}}\left({\bar{y}}_{h}^{\text{Ψ}}-\mu_{0}\right){\left({\bar{y}}_{h}^{\text{Ψ}}-\mu_{0}\right)}^{\text{T}}\right\}\right|}\right]}^{\frac{\nu_{0}+n}{2}}.$$

First note that for $n_{k_{n}}$, $n_{k_{n}-1}\geq 1$

(A.10)
$$\frac{\kappa_{0}\left(\kappa_{0}+n_{k_{n}-1}^{\prime }\right)}{\left(\kappa_{0}+n_{k_{n}-1}\right)\left(\kappa_{0}+n_{k_{n}}\right)}<1.$$

Similar to Corollary 2, we can assume without loss of generality $\mu_{0}$ to be a p-dimensional vector of zero and ${\text{Λ}}_{0}=I_{p}$. Note that,

(A.11)
$$\sum_{h=1}^{k_{n}}\;\left(S_{h}^{\text{Ψ}}+\frac{n_{h}\kappa_{0}}{n_{h}+\kappa_{0}}{\bar{y}}_{h}^{\text{Ψ}}{\bar{y}}_{h}^{{\text{Ψ}}^{\text{T}}}\right)=\sum_{i=1}^{n}\;y_{i}y_{i}^{\text{T}}-\sum_{h=1}^{k}\;\frac{n_{h}^{2}}{n_{h}+\kappa_{0}}{\bar{y}}_{h}^{\text{Ψ}}{\bar{y}}_{h}^{{\text{Ψ}}^{\text{T}}}\;\;=\sum_{i=1}^{n}\;y_{i}y_{i}^{\text{T}}-\sum_{h=1}^{k}\;\frac{1}{n_{h}+\kappa_{0}}\left(\sum_{i:c_{i}=h}y_{i}\right){\left(\sum_{i:c_{i}=h}y_{i}\right)}^{\text{T}}.$$

Also, without loss of generality we can assume $\left\{y_{1+\sum_{j=1}^{h-1}\;n_{j}},\ldots ,y_{\sum_{j=1}^{h}\;n_{j}}\right\}$ are in cluster h of Ψ similarly to Corollary 2. Then $\sum_{h=1}^{k_{n}}\;\left(S_{h}^{\text{Ψ}}+\frac{n_{h}\kappa_{0}}{n_{h}+\kappa_{0}}{\bar{y}}_{h}^{\text{Ψ}}{\bar{y}}_{h}^{{\text{Ψ}}^{\text{T}}}\right)=Y^{\text{T}}\left(I_{n}-J_{n}^{\text{Ψ}}\right)Y$, where $J_{n}^{\text{Ψ}}=\text{diag}\left(\frac{J_{n_{1}}}{n_{1}+\kappa_{0}},\ldots ,\frac{J_{n_{k_{n}}}}{n_{k_{n}}+\kappa_{0}}\right)$ is an n×n order block diagonal matrix and $J_{r}$ is the r×r order square matrix with all elements being 1. Clearly, $J_{n}^{\text{Ψ}}$ is a positive semi-definite matrix of rank $k_{n}$. Henceforth, exploiting the lower rank factorization structure, each determinant in (A.9) can be simplified as

$$\left|I_{p}+\sum_{h=1}^{k_{n}}\;\left(S_{h}^{\text{Ψ}}+\frac{n_{h}\kappa_{0}}{n_{h}+\kappa_{0}}{\bar{y}}_{h}^{\text{Ψ}}{\bar{y}}_{h}^{{\text{Ψ}}^{\text{T}}}\right)\right|=\left|I_{p}+Y^{\text{T}}\left(I_{n}-J_{n}^{\text{Ψ}}\right)Y\right|\;\;=\left|I_{p}+Y^{\text{T}}Y\right|\left|I_{n}-J_{n}^{\text{Ψ}1/2}Y{\left(I_{p}+Y^{\text{T}}Y\right)}^{-1}Y^{\text{T}}J_{n}^{{\text{Ψ}}^{1/2}}\right|.$$

Hence (A.9) reduces to

(A.12)
$${\left\{\frac{\kappa_{0}\left(\kappa_{0}+n_{k_{n}-1}^{\prime }\right)}{\left(\kappa_{0}+n_{k_{n}-1}\right)\left(\kappa_{0}+n_{k_{n}}\right)}\right\}}^{p/2}{\left\{\frac{\left|I_{n}-J_{n}^{{\text{Ψ}}^{\prime 1}/2}Y{\left(I_{p}+Y^{\text{T}}Y\right)}^{-1}Y^{\text{T}}J_{n}^{{\text{Ψ}}^{\prime 1}/2}\right|}{\left|I_{n}-J_{n}^{{\text{Ψ}}^{1/2}}Y{\left(I_{p}+Y^{\text{T}}Y\right)}^{-1}Y^{\text{T}}J_{n}^{{\text{Ψ}}^{1/2}}\right|}\right\}}^{\frac{\nu_{0}+n}{2}}.$$

From Lemma S.8 in the supplementary materials, $\lim_{p\to \infty }\;{\left\|Y{\left(I_{p}+Y^{\text{T}}Y\right)}^{-1}Y^{\text{T}}-I_{n}\right\|}_{2}=0$ in ${\mathbb{P}}_{0}^{p}$-probability. Therefore, from the construction of $J_{n}^{\text{Ψ}}$,

(A.13)
$$\underset{p\to \infty }{\lim}\;\left|I_{n}-J_{n}^{\text{Ψ}1/2}Y{\left(I_{p}+Y^{\text{T}}Y\right)}^{-1}Y^{\text{T}}J_{n}^{{\text{Ψ}}^{1/2}}\right|=\left|I_{n}-J_{n}^{\text{Ψ}}\right|=\prod_{h=1}^{k_{n}}\;\left|I_{n_{h}}-\frac{1}{n_{h}+\kappa_{0}}J_{n_{h}}\right|,$$

in ${\mathbb{P}}_{0}^{p}$-probability. Notably $J_{r}=1_{r}1_{r}^{\text{T}}$ where $1_{r}$ is the r-dimensional vector of ones, implying that $\left|I_{r}-\frac{1}{n_{r}+\kappa_{0}}J_{r}\right|=\frac{\kappa_{0}}{n_{r}+\kappa_{0}}$ for any positive integer r. Substituting this in (A.13), we have

$$\underset{p\to \infty }{\lim}\;\left|I_{n}-J_{n}^{\text{Ψ}1/2}Y{\left(I_{p}+Y^{\text{T}}Y\right)}^{-1}Y^{\text{T}}J_{n}^{\text{Ψ}1/2}\right|=\prod_{h=1}^{k_{n}}\;\frac{\kappa_{0}}{n_{h}+\kappa_{0}},\;\text{in}\;{\mathbb{P}}_{0}^{p}\text{-probability}$$

and therefore,

$$\underset{p\to \infty }{\lim}\;\frac{\left|I_{n}-J_{n}^{{\text{Ψ}}^{\prime 1}/2}Y{\left(I_{p}+Y^{\text{T}}Y\right)}^{-1}Y^{\text{T}}J_{n}^{{\text{Ψ}}^{\prime 1/2}}\right|}{\left|I_{n}-J_{n}^{{\text{Ψ}}^{1/2}}Y{\left(I_{p}+Y^{\text{T}}Y\right)}^{-1}Y^{\text{T}}J_{n}^{{\text{Ψ}}^{1/2}}\right|}=\frac{\left(\kappa_{0}+n_{k_{n}-1}\right)\left(\kappa_{0}+n_{k_{n}}\right)}{\kappa_{0}\left(\kappa_{0}+n_{k_{n}-1}^{\prime }\right)}\;\text{in}\;{\mathbb{P}}_{0}^{p}\text{-probability}.$$

Thus if we take the log of (A.12) multiplied by $p^{-1}$ and study its limit we have

$$\underset{p\to \infty }{\text{lim}\;\text{inf}}\;\left\{\frac{1}{2}\log\frac{\kappa_{0}\left(\kappa_{0}+n_{k_{n}-1}^{\prime }\right)}{\left(\kappa_{0}+n_{k_{n}-1}\right)\left(\kappa_{0}+n_{k_{n}}\right)}+\frac{n+\nu_{0}}{2p}\log\frac{\left|I_{n}-J_{n}^{{\text{Ψ}}^{\prime 1/2}}Y{\left(I_{p}+Y^{\text{T}}Y\right)}^{-1}Y^{\text{T}}J_{n}^{{\text{Ψ}}^{\prime 1/2}}\right|}{\left|I_{n}-J_{n}^{{\text{Ψ}}^{1/2}}Y{\left(I_{p}+Y^{\text{T}}Y\right)}^{-1}Y^{\text{T}}J_{n}^{{\text{Ψ}}^{1/2}}\right|}\right\}\;\;=\frac{1}{2}\log\frac{\kappa_{0}\left(\kappa_{0}+n_{k_{n}-1}^{\prime }\right)}{\left(\kappa_{0}+n_{k_{n}-1}\right)\left(\kappa_{0}+n_{k_{n}}\right)}\times \left(1-\underset{p\to \infty }{\text{lim}\;\text{sup}}\frac{n+\nu_{0}}{p}\right)>0.$$

Since n is fixed with p, the above limit follows from (A.10) and the assumption on $\nu_{0}$. Thus we have

$$\underset{p\to \infty }{\text{lim}\;\text{inf}}\;\frac{\int \prod_{h=1}^{k_{n}}\;\int \prod_{i:c_{i}=h}{\text{N}}_{p}\left(y_{i};\mu_{h},\text{Σ}\right){\text{N}}_{p}\left(\mu_{h};\mu_{0},\kappa_{0}^{-1}\text{Σ}\right)\text{d}\mu_{h}IW\left(\text{Σ};\nu_{0},{\text{Λ}}_{0}\right)\text{dΣ}}{\int \prod_{h=1}^{k_{n}-1}\;\int \prod_{i:c_{i}^{\prime }=h}{\text{N}}_{p}\left(y_{i};\mu_{h},\text{Σ}\right){\text{N}}_{p}\left(\mu_{h};\mu_{0},\kappa_{0}^{-1}\text{Σ}\right)\text{d}\mu_{h}IW\left(\text{Σ};\nu_{0},{\text{Λ}}_{0}\right)\text{dΣ}}=\infty ,$$

and hence for p→∞ each data point is clustered separately in ${\mathbb{P}}_{0}^{p}$-probability thanks to Theorem 1. ■

### Proofs of Section 4

**Proof** [Lemma 5] Let $\zeta_{0}^{\left(p\right)}={\left(\sqrt{p\log p}\right)}^{-1}{\left({\text{Λ}}^{\text{T}}\text{Λ}\right)}^{-1/2}{\text{Λ}}^{\text{T}}{\text{Λ}}_{0}\eta_{0}$, then $\text{Π}\left(\text{Ψ}|\eta_{0}\right)=\text{Π}\left(\text{Ψ}|\zeta_{0}^{\left(p\right)}\right)$. Then,

(A.14)
$$\frac{1}{\sqrt{p\log p}}\|\zeta_{i}^{\left(p\right)}-\zeta_{0i}^{\left(p\right)}\|\leq \|{\left({\text{Λ}}^{\text{T}}\text{Λ}\right)}^{-1/2}\text{Λ}{\|}_{2}\times \frac{1}{\sqrt{p\log p}}\|\text{Λ}\eta_{i}-{\text{Λ}}_{0}\eta_{0i}\|\leq \frac{1}{\sqrt{p\log p}}\|\text{Λ}\eta_{i}-{\text{Λ}}_{0}\eta_{0i}\|.$$

From (11) we see that the numerator in the right hand side of (10) can be simplified as

(A.15)
$$C\times \text{Π}(\text{Ψ})\times \prod_{h=1}^{k_{n}}\;{\left(\frac{\kappa_{0}}{n_{h}+\kappa_{0}}\right)}^{\frac{d}{2}}\times {\left|\sum_{h=1}^{k_{n}}\;\left\{S_{\eta_{0}}^{h}+\frac{n_{h}}{n_{h}+1}{\bar{\eta }}_{0}^{h}{\bar{\eta }}_{0}^{h^{\text{T}}}\right\}\right|}^{-\frac{n}{2}},$$

where $n_{h}=\sum_{i=1}^{n}\;I\left(c_{i}=h\right)$, ${\bar{\eta }}_{0}^{h}=\frac{1}{n_{h}}\sum_{i:c_{i}=h}\eta_{0i}$, $S_{\eta_{0}}^{h}=\sum_{i:c_{i}=h}\left(\eta_{0i}-{\bar{\eta }}_{0}^{h}\right){\left(\eta_{0i}-{\bar{\eta }}_{0}^{h}\right)}^{\text{T}}$ and C is a positive quantity constant across all ${\text{Ψ}}^{\prime }\in P$. Hence it is clear that Π(Ψ∣η) is a continuous function of η. Since the function is bounded (being a probability function), the continuity is also uniform. Also note that, for the particular choice of Gaussian kernel and base measure in (11), the oracle partition probability (10) is unchanged if η is multiplied by a full-rank square matrix and therefore $\text{Π}\left(\text{Ψ}|\zeta_{0}^{\left(p\right)}\right)=\text{Π}\left(\text{Ψ}|\eta_{0}\right)$. Therefore, for any ϵ>0 there exists δ>0 such that $\|\zeta_{0}^{\left(p\right)}-\zeta^{\left(p\right)}\|<\delta \;\text{implies that}\;\left|\text{Π}\left(\text{Ψ}|\zeta^{\left(p\right)}\right)-\text{Π}\left(\text{Ψ}|\zeta_{0}^{\left(p\right)}\right)\right|=\left|\text{Π}\left(\text{Ψ}|\zeta^{\left(p\right)}\right)-\text{Π}\left(\text{Ψ}|\eta_{0}\right)\right|<\epsilon$. Again,

(A.16)
$$E\left\{\left|\text{Π}\left(\text{Ψ}|\zeta^{\left(p\right)}\right)-\text{Π}\left(\text{Ψ}|\eta_{0}\right)\right||Y\right\}=E\left\{\left|\text{Π}\left(\text{Ψ}|\zeta^{\left(p\right)}\right)-\text{Π}\left(\text{Ψ}|\zeta_{0}^{\left(p\right)}\right)\right||B_{p,\delta },Y\right\}\text{Π}\left(B_{p,\delta }|Y\right)\;\;+E\left\{\left|\text{Π}\left(\text{Ψ}|\zeta^{\left(p\right)}\right)-\text{Π}\left(\text{Ψ}|\eta_{0}\right)\right||{\bar{B}}_{p,\delta },Y\right\}\text{Π}\left({\bar{B}}_{p,\delta }|Y\right).$$

Due to continuity, δ can be chosen sufficiently small such that the term inside the first expectation in the right hand side of (A.16) is smaller than arbitrarily small ϵ>0. Now for any δ>0, the second term in the right hand side of (A.16) goes to 0 as $\text{Π}\left({\bar{B}}_{p,\delta }|Y\right)\to 0$ as p→∞ by assumption. Therefore, for arbitrarily small ϵ>0, $E\left\{\left|\text{Π}\left(\text{Ψ}|\zeta^{\left(p\right)}\right)-\text{Π}\left(\text{Ψ}|\eta_{0}\right)\right||Y\right\}<\epsilon$ for large enough p. Hence the proof. ■
