# Supplementary material for: Escaping The Curse of Dimensionality in Bayesian Model-Based Clustering
Source: J Mach Learn Res. Author manuscript; Available in PMC 2025 Apr 15. (PMC11999651)
Supplement: 1 [file NIHMS1925507-supplement-1.pdf]

Supplementary Materials for **Escaping the Curse of Dimensionality in Bayesian Model-based Clustering**

The supplementary materials present proofs of some theoretical results, simulation studies and MCMC convergence diagnostics.

S.1. Additional Theoretical Results

In the supplementary materials, we denote by $\|x\|$ the Euclidean norm of a vector x and by $\|X\|_2$ the spectral norm of a matrix X. The smallest and largest eigenvalues of the matrix $(X^{\mathrm{T}}X)^{\frac{1}{2}}$ are denoted by $s_{\min}(X)$ and $s_{\max}(X)$, respectively. For a positive-definite matrix X, $\lambda_{\min}(X)$ and $\lambda_{\max}(X)$ denote the smallest and largest eigenvalues, respectively.

Lemma S.7 *Let $\Gamma_p(\cdot)$ be the multivariate gamma function, $\nu_0 = p+c$ for some constant $c \geq 0$, and ℓ and m be (not varying with p) non-negative integers. Then,* $\lim_{p\to\infty} \frac{1}{p} \log\{\Gamma_p(\frac{\nu_0+\ell}{2})/\Gamma_p(\frac{\nu_0+m}{2})\} = 0$.

Proof Without loss of generality assuming $\ell > m$, we have

$$\frac{\Gamma_p(\frac{\nu_0+\ell}{2})}{\Gamma_p(\frac{\nu_0+m}{2})} = \prod_{j=1}^{p} \frac{\Gamma\left(\frac{\nu_0+\ell-j+1}{2}\right)}{\Gamma\left(\frac{\nu_0+m-j+1}{2}\right)} = \frac{\prod_{j=m+1}^{\ell} \Gamma\left(\frac{\nu_0+j}{2}\right)}{\prod_{j=m}^{\ell} \Gamma\left(\frac{\nu_0+j-p}{2}\right)}. \tag{S.1}$$

Note that the denominator term in the extreme right hand of (S.1) does not depend on p as $\nu_0 - p$ is constant from assumption. Applying Stirling's approximation on the numerator we get

$$\begin{aligned}\frac{\Gamma_p(\frac{\nu_0+\ell}{2})}{\Gamma_p(\frac{\nu_0+m}{2})} &= \frac{1}{\prod_{j=m}^{\ell} \Gamma\left(\frac{\nu_0+j-p}{2}\right)} \times \prod_{j=m+1}^{\ell} \left\{ \sqrt{2\pi\frac{\nu_0+j-1}{2}} \left(\frac{\nu_0+j-1}{2e}\right)^{\frac{\nu_0+j-1}{2}} E_j \right\} \\ &= \frac{1}{\prod_{j=m}^{\ell} \Gamma\left(\frac{\nu_0+j-p}{2}\right)} \times \prod_{j=m+1}^{\ell} \left\{ \sqrt{2\pi e} \left(\frac{\nu_0}{2e}\right)^{\frac{\nu_0+j+1}{2}} \left(1+\frac{j-1}{\nu_0}\right)^{\frac{\nu_0+j+1}{2}} E_j \right\},\end{aligned}$$

where $E_j = O(\log p)$ arising from the Stirling's approximation formulae. Using the result $\lim_{x\to\infty}(1+c/x)^x = e^c$, it can be seen that

$$\begin{aligned}&\lim_{p\to\infty} \prod_{j=m+1}^{\ell} \left\{ \sqrt{2\pi e} \left(\frac{\nu_0}{2e}\right)^{\frac{\nu_0+j+1}{2}} \left(1+\frac{j-1}{\nu_0}\right)^{\frac{\nu_0+j+1}{2}} \right\} \\ &\qquad = (2\pi e)^{\frac{\ell-m}{2}} \times \left(\frac{\nu_0}{2e}\right)^{\frac{1}{4}(\ell-m)(2\nu_0+m+\ell+3)} \times e^{\frac{1}{2}(\ell-m)(\ell+m-1)},\end{aligned}$$

which is a finite quantity. Hence the proof. ■

Lemma S.8 *For any $n \times p$ order matrix Y satisfying (A0),* $\lim_{p\to\infty} \left\|Y(I_p + Y^{\mathrm{T}}Y)^{-1}Y^{\mathrm{T}} - I_n\right\|_2 = 0$ *in $\mathbb{P}_0^p$-probability.*

Proof Letting $Y = UDV$, the singular value decomposition of Y, we have $Y(I_p + Y^{\mathrm{T}}Y)^{-1}Y^{\mathrm{T}} = U\,\mathrm{diag}\left(\frac{d_1^2}{1+d_1^2}, \ldots, \frac{d_n^2}{1+d_n^2}\right) U^{\mathrm{T}}$ where $d_1, \ldots, d_n$ are the singular values of Y in descending order. From (A0) we have $\liminf_{p\to\infty} \frac{1}{p} d_i^2 > 0$, which further implies that $\liminf_{p\to\infty} \frac{d_i}{1+d_i} \to 1$ for all $i = 1, \ldots, n$. As $\frac{d_i}{1+d_i} \leq 1$, $\lim_{p\to\infty} \left\|Y(I_p + Y^{\mathrm{T}}Y)^{-1}Y^{\mathrm{T}} - I_n\right\|_2 = 0$ in $\mathbb{P}_0^p$-probability. ■

Lemma S.9 *Let $\widetilde{Y}$ be an $\widetilde{n} \times p$ order matrix, formed by arbitrarily selecting $\widetilde{n}$ rows from $Y = [y_1, \ldots, y_n]^{\mathrm{T}}$ where $1 \leq \widetilde{n} \leq n$. If Y satisfies (A0), then* $\lim_{p\to\infty} \left\|\widetilde{Y}(I_p + \widetilde{Y}^{\mathrm{T}}\widetilde{Y})^{-1}\widetilde{Y}^{\mathrm{T}} - I_{\widetilde{n}}\right\|_2 = 0$ *in $\mathbb{P}_0^p$-probability.*

Proof Letting $Y = UDV$ the singular value decomposition of Y, we have $\widetilde{Y} = \widetilde{U}DV$ where $\widetilde{U}$ is formed by the corresponding rows of Y which were used to form $\widetilde{Y}$. Using Pati et al. (2014, Lemma 1.1(iii) from the Suppplementary section), we have $s_{\min}(\widetilde{Y}^{\mathrm{T}}) \geq s_{\min}(V^{\mathrm{T}})s_{\min}(D^{\mathrm{T}})s_{\min}(\widetilde{U}^{\mathrm{T}}) = s_{\min}(Y^{\mathrm{T}})$. Since $s_{\min}(\widetilde{U}^{\mathrm{T}}) = s_{\min}(U^{\mathrm{T}}) = 1$, $s_{\min}(\widetilde{Y}^{\mathrm{T}}) \geq s_{\min}(Y^{\mathrm{T}})$. Therefore, $\widetilde{Y}$ also satisfies (A0) if we substitute $Y = \widetilde{Y}$. Consequently applying Lemma S.8, we conclude the proof. ■

Lemma S.10 *Let Y be an $n \times p$ order matrix satisfying (A0). Let $Y_i = [y_{j_{i,1}}, \ldots, y_{j_{i,n_i}}]^{\mathrm{T}}$, $i = 1, 2$ be an arbitrary partiton of the data-matrix into two sub-matrices such that $n_1 + n_2 = n$. Then* $\limsup_{p\to\infty} s_{\max}(Z) < 1$ *where* $Z = (I_{n_1} + Y_1Y_1^{\mathrm{T}})^{-1/2}Y_1Y_2^{\mathrm{T}}(I_{n_2} + Y_2Y_2^{\mathrm{T}})^{-1/2}$ *in $\mathbb{P}_0^p$-probability.*

Proof From (A0) we have $\left\|YY^{\mathrm{T}}\right\|_2 = O(p)$ and $\liminf \lambda_{\min}(YY^{\mathrm{T}})/p > 0$, which implies that

$$0 < \liminf_{p\to\infty} \left|(I_n + YY^{\mathrm{T}})/p\right| \leq \limsup_{p\to\infty} \left|(I_n + YY^{\mathrm{T}})/p\right| = O(1) \text{ in } \mathbb{P}_0^p\text{-probability.} \tag{S.2}$$

Following the proof of Lemma S.9, we see that Y_i also satisfies (A0), and therefore (S.2) also holds if Y is replaced with Y_i for $i = 1, 2$. Noting that $I_n + YY^{\mathrm{T}} = \begin{bmatrix} I_{n_1} + Y_1Y_1^{\mathrm{T}} & Y_1Y_2^{\mathrm{T}} \\ Y_2Y_1^{\mathrm{T}} & I_{n_2} + Y_2Y_2^{\mathrm{T}} \end{bmatrix}$ and using matrix factorization results, we have

$$\left|\frac{1}{p}(I_n + YY^{\mathrm{T}})\right| = \left|\frac{1}{p}(I_{n_1} + Y_1Y_1^{\mathrm{T}})\right|\left|\frac{1}{p}(I_{n_2} + Y_2Y_2^{\mathrm{T}})\right|\left|I_{n_1} - ZZ^{\mathrm{T}}\right|. \tag{S.3}$$

Again in $\mathbb{P}_0^p$-probability,

$$\limsup_{p\to\infty} s_{\max}^2(Z) \leq \limsup_{p\to\infty} \left\|Y_1^{\mathrm{T}}(I_{n_1} + Y_1Y_1^{\mathrm{T}})^{-1}Y_1\right\|_2 \left\|Y_2^{\mathrm{T}}(I_{n_2} + Y_2Y_2^{\mathrm{T}})^{-1}Y_2\right\|_2 \leq 1. \tag{S.4}$$

For (S.2) to hold, all the terms in the RHS of (S.3) must be bounded away from 0. As $\left|I_{n_1} - ZZ^{\mathrm{T}}\right| = \prod_{j=1}^{n_1} \left|1 - s_j^2(Z)\right|$, the inequality on (S.4) must be strict. Thus, we conclude the proof. ■

Lemma S.11 *For prior* (7) $\lim_{p\to\infty} \frac{1}{p}\lambda_{\min}(\Lambda^{\mathrm{T}}\Lambda) = \lim_{p\to\infty} \frac{1}{p}\lambda_{\max}(\Lambda^{\mathrm{T}}\Lambda) = v_1$ *for some* $v_1 > 0$ Π-*a.s.*

Proof From Bhattacharya et al. (2015, Eqn (9) and Section 2.4) we get that $\Lambda^{\mathrm{T}}\Lambda = \tau^2 T^{\mathrm{T}} T$ where the (i, j)-th element of T is $t_{ij} = e_{ij}\phi_{ij}$ with $e_{ij} \overset{iid}{\sim} \text{DE}(1)$ where $\text{DE}(b)$ is the double exponential distribution with median 0 and variance $2b^2$. Additionally $\phi \sim \text{Dir}(a, \ldots, a)$ and $\tau \sim \text{Ga}(pda, 1/2)$.

Now by the strong law of large numbers $\left\| \frac{1}{p}\tilde{T}^{\mathrm{T}}\tilde{T} - v_1 I_d \right\|_F \to 0$ as $p \to \infty$ where $\|\cdot\|_F$ is the Frobenius norm of a matrix and $v_1 = \text{Var}(e_{ij}\gamma_{ij})$. Hence, for any $i = 1, \ldots, p$ $\lim_{p\to\infty} \lambda_i(\tilde{T}^{\mathrm{T}}\tilde{T})p^{-1} = v_1$. Also $\lim_{p\to\infty} \tau/(pd) = E(\tau_{ij})$ which implies that $\lim_{p\to\infty} (\tau/\Gamma)^2 = 1$ Π-a.s. Hence the proof. ■

S.2. Proof of Theorem 6 and Associated Results

To prove Theorem 6, we consider an adaptation of Theorem 6.39 in Ghosal and Van Der Vaart (2017) where instead of having an increasing sample size, we assume an increasing data dimension with fixed sample size. This notion is consistent with the idea that more and more variables are measured on each study subject. We introduce the following notation. Let $\vartheta = (\Lambda, \eta, \sigma)$ with $\eta = [\eta_1, \ldots, \eta_n]^{\mathrm{T}}$ and $\vartheta \in \Theta_p$. Let $\mathbb{P}^p_\vartheta$ and $\mathbb{P}^p_0$ be the joint distributions of the data $y_1, \ldots, y_n$ given ϑ and ϑ_0, respectively, with $\vartheta_0 = (\Lambda_0, \eta_0, \sigma_0)$. We also denote the expectation of a function g with respect to $\mathbb{P}^p_0$ and $\mathbb{P}^p_\vartheta$ by $\mathbb{P}^p_0 g$ and $\mathbb{P}^p_\vartheta g$ respectively. Let p^p_0 and p^p_ϑ be the densities of $\mathbb{P}^p_0$ and $\mathbb{P}^p_\vartheta$ with respect to the Lebesgue measure. Finally, define the Kullback-Leibler (KL) divergence and the r-th order positive KL-variation between p^p_0 and p^p_ϑ, respectively, as $KL(\mathbb{P}^p_0, \mathbb{P}^p_\vartheta) = \int \log \frac{p^p_0}{p^p_\vartheta} \mathrm{d}\mathbb{P}^p_0$ and $V^+_r(\mathbb{P}^p_0, \mathbb{P}^p_\vartheta) = \int \left\{ \left(\log \frac{p^p_0}{p^p_\vartheta} - KL \right)^+ \right\}^r \mathrm{d}\mathbb{P}^p_0$, where f^+ denotes the positive part of a function f.

Theorem S.12 *If for some* $r \geq 2$, $c > 0$ *there exist measurable sets* $B_p \subset \Theta_p$ *with* $\liminf \Pi(B_p) > 0$,

(I) $\sup_{\vartheta \in B_p} \frac{1}{p} KL(\mathbb{P}^p_0, \mathbb{P}^p_\vartheta) \leq c$ *and* $\sup_{\vartheta \in B_p} \frac{1}{p^r} V^+_r(\mathbb{P}^p_0, \mathbb{P}^p_\vartheta) \to 0$.

(II) For sets $\tilde{\Theta}_p \subset \Theta_p$ *there exists a sequence of test functions* ϕ_p *such that* $\phi_p \to 0$ $\mathbb{P}^p_0$-*a.s. and* $\int_{\tilde{\Theta}_p} \mathbb{P}^p_\vartheta(1 - \phi_p)\mathrm{d}\Pi(\vartheta) \leq e^{-Cp}$ *for some* $C > 0$.

(III) Letting $A_p = \left\{ \vartheta \in \Theta_p : \frac{1}{p} \int \left(\log \frac{p^p_0}{p^p_\vartheta} - KL(\mathbb{P}^p_0, \mathbb{P}^p_\vartheta) \right) \mathrm{d}\tilde{\Pi}_p(\vartheta) < \tilde{\epsilon} \right\}$, *with* $\tilde{\Pi}_p$ *the renormalized restriction of* Π *to set* B_p, *for any* $\tilde{\epsilon} > 0$, $\mathbb{1}(\bar{A}_p) \to 0$ $\mathbb{P}^p_0$-*a.s.*

Then $\Pi(\tilde{\Theta}_p \mid \mathcal{Y}) \to 0$ $\mathbb{P}^p_0$-*a.s.*

Condition (I) ensures that the assumed model is not too far from the true data-generating model. Condition (II) controls the variability of the log-likelihood around its mean. In the Lamb model, the number of parameters grows with p and hence the assumption on V^+_r is instrumental. The conditions on ϕ_p ensure the existence of a sequence of consistent test functions for $H_0 : \mathbb{P} = \mathbb{P}^p_0$ in which type-II error diminishes to 0 exponentially fast in the critical region. Condition (III) is

a technical condition required to bound the numerator of $\Pi(\tilde{\Theta}_p \mid \mathcal{Y})$. The proof of this theorem follows along the lines of the proof of Theorem 6.39 of Ghosal and Van Der Vaart (2017).

Theorem S.12 is a general result stating sufficient conditions for posterior consistency as $p \to \infty$. We use this theorem to prove Theorem 6.

Proof [Theorem 6] We verify the conditions (I)-(III) from Theorem S.12. Theorems S.13 and S.14 jointly imply that for the Lamb model there exist a sequence of sets B_p such that conditions (I) and (III) are satisfied for any $c > 0$. Theorem S.15 ensures the existence of a sequence of test functions satisfying (II), and finally Theorem S.16 proves (III). Hence the proof. ∎

Theorem S.13 *For any $\epsilon > 0$ define $B_p^\epsilon = \left\{\Theta : p^{-1}KL(\mathbb{P}_0^p, \mathbb{P}_\vartheta^p) \leq \epsilon\right\}$. Then, under the settings of Section 4,* $\liminf \Pi(B_p^\epsilon) > 0$.

Proof Let P_0 and P be p-variate multivariate normal distributions with $P = \mathrm{N}_p(\mu, \Sigma)$ and $P_0 = \mathrm{N}_p(\mu_0, \Sigma_0)$. Then their Kullback-Leibler divergence is $KL(P_0, P) = \frac{1}{2}\{\log \frac{|\Sigma|}{|\Sigma_0|} + \mathrm{tr}\left(\Sigma^{-1}\Sigma_0\right) + (\mu - \mu_0)^{\mathrm{T}}\Sigma^{-1}(\mu - \mu_0) - p\}$, which, under the settings of Section 4, simplifies to

$$KL(\mathbb{P}_0^p, \mathbb{P}_\vartheta^p) = \frac{1}{2}\left\{np \log \frac{\sigma^2}{\sigma_0^2} + np\left(\frac{\sigma_0^2}{\sigma^2} - 1\right) + \frac{1}{\sigma^2}\sum_{i=1}^n \|\mu_i - \mu_{0i}\|^2\right\}, \tag{S.5}$$

where $\mu_i = \Lambda\eta_i$ and $\mu_{0i} = \Lambda_0\eta_{0i}$. Now,

$$\begin{aligned}\Pi\left\{p^{-1}KL(\mathbb{P}_0^p, \mathbb{P}_\vartheta^p) < \epsilon\right\} &= \Pi\left\{n \log \frac{\sigma^2}{\sigma_0^2} + n\left(\frac{\sigma_0^2}{\sigma^2} - 1\right) + \frac{1}{p\sigma^2}\sum_{i=1}^n \|\mu_i - \mu_{0i}\|^2 < \epsilon\right\} \\ &\geq \Pi\left\{\log \frac{\sigma^2}{\sigma_0^2} + \left(\frac{\sigma_0^2}{\sigma^2} - 1\right) \leq \frac{\epsilon}{2n}, \frac{1}{\sigma^2}\sum_{i=1}^n \|\mu_i - \mu_{0i}\|^2 < \frac{p\epsilon}{2}\right\}.\end{aligned}$$

Note that for any $x > 0$, $\log x \leq x - 1$ and therefore $\log \frac{\sigma^2}{\sigma_0^2} + \left(\frac{\sigma_0^2}{\sigma^2} - 1\right) \leq \left(\frac{\sigma_0}{\sigma} - \frac{\sigma}{\sigma_0}\right)^2$ implying that

$$\begin{aligned}\Pi\left\{p^{-1}KL(\mathbb{P}_0^p, \mathbb{P}_\vartheta^p) < \epsilon\right\} &\geq \Pi\left\{\left(\frac{\sigma_0}{\sigma} - \frac{\sigma}{\sigma_0}\right)^2 \leq \frac{\epsilon}{2n}, \frac{1}{\sigma^2}\sum_{i=1}^n \|\mu_i - \mu_{0i}\|^2 < \frac{p\epsilon}{2}\right\} \\ &\geq \Pi\left\{\left(\frac{\sigma_0}{\sigma} - \frac{\sigma}{\sigma_0}\right)^2 \leq \frac{\epsilon}{2n}\right\}\Pi\left(\sum_{i=1}^n \|\mu_i - \mu_{0i}\|^2 < \sigma_L\frac{p\epsilon}{2}\right),\end{aligned}$$

where the second inequality holds thanks to condition (C3). The first factor above is positive under our proposed prior on σ. Now consider the second factor and note that for each $i = 1, \ldots, n$, $\|\mu_i - \mu_{0i}\|^2 = \left\|\Lambda(\eta_i - (\Lambda^{\mathrm{T}}\Lambda)^{-1}\Lambda^{\mathrm{T}}\Lambda_0\eta_{0i})\right\|^2 + \eta_{0i}^{\mathrm{T}}(\Lambda_0^{\mathrm{T}}\Lambda_0 - \Lambda_0^{\mathrm{T}}\Lambda(\Lambda^{\mathrm{T}}\Lambda)^{-1}\Lambda^{\mathrm{T}}\Lambda_0)\eta_{0i}$. By the triangle inequality

$$\frac{1}{p}\left\|\Lambda_0^{\mathrm{T}}\Lambda_0 - \Lambda_0^{\mathrm{T}}\Lambda(\Lambda^{\mathrm{T}}\Lambda)^{-1}\Lambda^{\mathrm{T}}\Lambda_0\right\|_2 \leq \left\|\frac{1}{p}\Lambda_0^{\mathrm{T}}\Lambda_0 - M\right\|_2 + \left\|M - \frac{1}{p}\Lambda_0^{\mathrm{T}}\Lambda(\Lambda^{\mathrm{T}}\Lambda)^{-1}\Lambda^{\mathrm{T}}\Lambda_0\right\|_2. \tag{S.6}$$

The first term on the right hand side of (S.6) goes to 0 as $p \to \infty$ by (C2). Let us define the matrix $B = (\Lambda^{\mathrm{T}}\Lambda)^{-1/2}\Lambda^{\mathrm{T}}$, with $\|B\|_2 = 1$ and $\tilde{\Lambda}_0 = \Lambda_0 M^{-1/2}$ where $M^{1/2}$ is the Cholesky factor of M. From Vershynin (2012, Theorem 5.39) it follows that for any $0 < \epsilon < 1$ and large enough p, $1-\epsilon \le \left\|\frac{1}{\sqrt{p}}\tilde{\Lambda}_0\right\|_2 \le 1+\epsilon$. Again, from Lemma 1.1 of the Supplement section of Pati et al. (2014) we have that $1-\epsilon \le \left\|\frac{1}{\sqrt{p}}B\tilde{\Lambda}_0\right\|_2 \le 1+\epsilon$, $\quad 1-\epsilon \le \frac{1}{\sqrt{p}}s_{\min}(B\tilde{\Lambda}_0) \le 1+\epsilon$. Therefore $\lim_{p\to\infty}\lambda_i(\tilde{\Lambda}_0^{\mathrm{T}}\Lambda(\Lambda^{\mathrm{T}}\Lambda)^{-1}\Lambda^{\mathrm{T}}\tilde{\Lambda}_0)p^{-1} = 1$ for all $i = 1,\ldots,d$. Now $\left\|M - \frac{1}{p}\Lambda_0^{\mathrm{T}}\Lambda(\Lambda^{\mathrm{T}}\Lambda)^{-1}\Lambda^{\mathrm{T}}\Lambda_0\right\|_2 = \|M\|_2\left\|I_d - \frac{1}{p}\tilde{\Lambda}_0^{\mathrm{T}}\Lambda(\Lambda^{\mathrm{T}}\Lambda)^{-1}\Lambda^{\mathrm{T}}\tilde{\Lambda}_0\right\|_2$ and therefore the second term on the right hand side of (S.6) goes to 0 as $p\to\infty$. Subsequently we have $\lim_{p\to\infty}\frac{1}{p}\eta_{0i}^{\mathrm{T}}(\Lambda_0^{\mathrm{T}}\Lambda_0 - \Lambda_0^{\mathrm{T}}\Lambda(\Lambda^{\mathrm{T}}\Lambda)^{-1}\Lambda^{\mathrm{T}}\Lambda_0)\eta_{0i} = 0$ for all $i = 1,\ldots,n$. Now (C2) and Lemma S.11 jointly imply that $\left\|(\Lambda^{\mathrm{T}}\Lambda)^{-1}\Lambda^{\mathrm{T}}\Lambda_0\right\|_2 = O(1)$ Π-a.s. Therefore, for standard normal priors on the latent variables, $\liminf_{p\to\infty}\Pi(\sum_{i=1}^n\|\mu_i - \mu_{0i}\|^2 < \sigma_L p\epsilon) > 0$. From the permanence of KL-property of mixture priors (Ghosal and Van Der Vaart, 2017, Proposition 6.28) we can conclude that the right hand side is also positive. ■

Theorem S.14 *On the set B_p^ϵ defined in Theorem S.13, we have $V_r^+(\mathbb{P}_0^p,\mathbb{P}_\vartheta^p) = o(p^r)$ for $r = 2$.*

Proof For $r = 2$, $V_r^+(\mathbb{P}_0^p,\mathbb{P}_\vartheta^p) \le \int \log^2\frac{p_0^p}{p_\vartheta^p}d\mathbb{P}_0^p - \left\{\int\log\frac{p_0^p}{p_\vartheta^p}d\mathbb{P}_0^p\right\}^2$. Now conditionally on $\vartheta\in\vartheta$, the observations $y_1,\ldots,y_n$ are independent. Therefore,

$$V_r^+(\mathbb{P}_0^p,\mathbb{P}_\vartheta^p) \le \sum_{j=1}^n\left[\int\left\{\log\frac{p_{0j}(y_j)}{p_{\vartheta_j}(y_j)}\right\}^2 p_{0j}(y_j)\mathrm{d}y_j - \left\{\int\log\frac{p_{0j}(y_j)}{p_{\vartheta_j}(y_j)}p_{0j}(y_j)\mathrm{d}y_j\right\}^2\right] \tag{S.7}$$

where $p_{0j}(y_j) = \prod_{i=1}^p \mathrm{N}(y_{ji};\mu_{0ji},\sigma_0^2)$ and $p_{\vartheta_j}(y_j) = \prod_{i=1}^p\mathrm{N}(y_{ji};\mu_{ji},\sigma^2)$ with $\mu_{0j} =$ and $\mu_j = \Lambda\eta_j$. We first show the result for a particular term inside the summation of (S.7). Since $\|\eta_{0i}\| = O(1)$ and n is fixed, the result will readily follow afterwards. For simplicity, we drop the suffix j from the terms of (S.7) henceforth. Consider,

$$\begin{aligned}\left\{\log\frac{p_0(y_i)}{p_\vartheta(y_i)}\right\}^2 &= \left[\log\frac{\sigma}{\sigma_0} - \frac{1}{2}\left\{\left(\frac{y_i-\mu_{0i}}{\sigma_0}\right)^2 - \left(\frac{y_i-\mu_i}{\sigma}\right)^2\right\}\right]^2\\ &= \frac{1}{4}\left\{\left(\frac{y_i-\mu_{0i}}{\sigma_0}\right)^2 - \left(\frac{y_i-\mu_i}{\sigma}\right)^2\right\}^2 + \log^2\frac{\sigma}{\sigma_0} - \left\{\left(\frac{y_i-\mu_{0i}}{\sigma_0}\right)^2 - \left(\frac{y_i-\mu_i}{\sigma}\right)^2\right\}\log\frac{\sigma}{\sigma_0}.\end{aligned}$$

Note that,

$$\begin{aligned}\left\{\left(\frac{y_i-\mu_{0i}}{\sigma_0}\right)^2 - \left(\frac{y_i-\mu_i}{\sigma}\right)^2\right\}^2 &= \left\{z_i^2\left(1-\frac{\sigma_0^2}{\sigma^2}\right) - 2z_i(\mu_{0i}-\mu_i)\frac{\sigma_0}{\sigma} + \left(\frac{\mu_i-\mu_{0i}}{\sigma}\right)^2\right\}^2\\ &= z_i^4\left(1-\frac{\sigma_0^2}{\sigma^2}\right)^2 + 4z_i^2\sigma_0^2\left(\frac{\mu_{0i}-\mu_i}{\sigma}\right)^2 + \left(\frac{\mu_i-\mu_{0i}}{\sigma}\right)^4 - 2z_i^3\left(1-\frac{\sigma_0^2}{\sigma^2}\right)\frac{\sigma_0}{\sigma}(\mu_{0i}-\mu_i)\\ &\quad - 2z_i\sigma_0\left(\frac{\mu_{0i}-\mu_i}{\sigma}\right)^3 + 2z_i^2\left(\frac{\mu_{0i}-\mu_i}{\sigma}\right)^2\left(1-\frac{\sigma_0^2}{\sigma^2}\right)\end{aligned}$$

where $z_i = (y_i - \mu_{0i})/\sigma_0$ and $z_i \stackrel{\text{iid}}{\sim} \mathrm{N}(0,1)$. Therefore,

$$E_{y_i}\left\{\left(\frac{y_i-\mu_{0i}}{\sigma_0}\right)^2 - \left(\frac{y_i-\mu_i}{\sigma}\right)^2\right\} = \left(1-\frac{\sigma_0^2}{\sigma^2}\right) + \left(\frac{\mu_i-\mu_{0i}}{\sigma}\right)^2 \text{ and}$$

$$E_{y_i}\left\{\left(\frac{y_i-\mu_{0i}}{\sigma_0}\right)^2 - \left(\frac{y_i-\mu_i}{\sigma}\right)^2\right\}^2 = 3\left(1-\frac{\sigma_0^2}{\sigma^2}\right)^2 + 4\sigma_0^2\left(\frac{\mu_{0i}-\mu_i}{\sigma}\right)^2 + \left(\frac{\mu_i-\mu_{0i}}{\sigma}\right)^4 + 2\left(\frac{\mu_{0i}-\mu_i}{\sigma}\right)^2\left(1-\frac{\sigma_0^2}{\sigma^2}\right).$$

Hence,

$$\int\left\{\log\frac{p_0(y_i)}{p_\vartheta(y_i)}\right\}^2 p_0(y_i)\mathrm{d}y_i = \left(\frac{\mu_{0i}-\mu_i}{\sigma}\right)^2 \times \left\{\sigma_0^2 + \frac{1}{2}\left(1-\frac{\sigma_0^2}{\sigma^2}\right) - \log\frac{\sigma}{\sigma_0}\right\} - \log\frac{\sigma}{\sigma_0}\left(1-\frac{\sigma_0^2}{\sigma^2}\right) + \frac{1}{4}\left(\frac{\mu_{0i}-\mu_i}{\sigma}\right)^4 + \frac{3}{4}\left(1-\frac{\sigma_0^2}{\sigma^2}\right)^2 + \log^2\frac{\sigma}{\sigma_0}$$

$$\left\{\int\log\frac{p_0(y_i)}{p_\vartheta(y_i)}p_0(y_i)\mathrm{d}y_i\right\}^2 = \left\{\log\frac{\sigma}{\sigma_0} + \frac{\sigma_0^2+(\mu_{0i}-\mu_i)^2}{2\sigma^2} - \frac{1}{2}\right\}^2 = \log^2\frac{\sigma}{\sigma_0} + \frac{1}{4}\left(1-\frac{\sigma_0^2}{\sigma^2}\right)^2 + \frac{1}{4}\left(\frac{\mu_{0i}-\mu_i}{\sigma}\right)^4 + \left(\frac{\mu_{0i}-\mu_i}{\sigma}\right)^2 \times \left\{\log\frac{\sigma}{\sigma_0} - \frac{1}{2}\left(1-\frac{\sigma_0^2}{\sigma^2}\right)\right\} - \log\frac{\sigma}{\sigma_0}\left(1-\frac{\sigma_0^2}{\sigma^2}\right),$$

leading to

$$\begin{aligned} V_r^+(\mathbb{P}_0^p, \mathbb{P}_\vartheta^p) &\le \sum_{i=1}^p\left[\int\left\{\log\frac{p_0(y_i)}{p_\vartheta(y_i)}\right\}^2 p_0(y_i)\mathrm{d}y_i - \left\{\int\log\frac{p_0(y_i)}{p_\vartheta(y_i)}p_0(y_i)\mathrm{d}y_i\right\}^2\right] \\ &= \frac{p}{2}\left(1-\frac{\sigma_0^2}{\sigma^2}\right)^2 + \left\{\sigma_0^2 - 2\log\frac{\sigma}{\sigma_0} + \left(1-\frac{\sigma_0^2}{\sigma^2}\right)\right\} \times \sum_{i=1}^p\left(\frac{\mu_{0i}-\mu_i}{\sigma}\right)^2. \end{aligned} \tag{S.8}$$

Note that

$$\sum_{i=1}^p(\mu_{0i}-\mu_i)^2 = \sum_{i=1}^p\left(\lambda_{0i}^{\mathrm{T}}\eta_0 - \lambda_i^{\mathrm{T}}\eta\right)^2 = \eta_0^{\mathrm{T}}\Lambda_0^{\mathrm{T}}\Lambda_0\eta_0 + \eta^{\mathrm{T}}\Lambda^{\mathrm{T}}\Lambda\eta - 2\eta_0^{\mathrm{T}}\Lambda_0^{\mathrm{T}}\Lambda\eta. \tag{S.9}$$

Now $\eta_0^{\mathrm{T}}\Lambda_0^{\mathrm{T}}\Lambda_0\eta_0 \le \|\Lambda_0\|_2^2\|\eta_0\|^2$ and therefore, by conditions (C2) and (C4), $\eta_0^{\mathrm{T}}\Lambda_0^{\mathrm{T}}\Lambda_0\eta_0 = O(p)$. Also from Lemma S.11, $\frac{1}{p}\|\Lambda\|_2^2 \le c$ for large enough p and some $c > 0$ and therefore $\eta^{\mathrm{T}}\Lambda^{\mathrm{T}}\Lambda\eta \le \|\Lambda\|_2^2\|\eta\|^2 = \|\eta\|^2 O(p)$. From the proof of Theorem S.13 we can see that in the set B_p^ϵ, $\|\eta\|$ is bounded. We have shown that the highest powers in (S.9) and thus in (S.8) are almost surely bounded by p for large enough p. Hence the proof. ■

Theorem S.15 *Let us define the test function* $\phi_p = \mathbb{1}\left\{\left|\frac{1}{\sqrt{np}\sigma_0}\|\sum_{i=1}^n(y_i - \Lambda_0\eta_{0i})\| - 1\right| > \tau\right\}$ *to test the following hypothesis* $H_0 : y_1, \ldots, y_n \sim \mathbb{P}_0^p$ *versus* $H_1 : H_0$ *is false where* τ *is a positive real number. Define the set* $\tilde{\Theta}_p = \bar{B}_{p,\delta}$*. Then there exists a constant* $C > 0$ *such that* $\phi_p \to 0$ $\mathbb{P}_0^p$*-a.s. and* $\int_{\tilde{\Theta}_p} \mathbb{P}_\vartheta^p(1-\phi_p)\mathrm{d}\Pi(\vartheta) \leq e^{-Cp}$*.*

Proof Let us define $\mu_i = \Lambda\eta_i$ and $\mu_{0i} = \Lambda\eta_{0i}$. Then under H_0, $\frac{1}{\sqrt{n}\sigma_0}\sum_{i=1}^n(y_i - \Lambda\eta_{0i}) \sim \mathrm{N}_p(0, I_p)$ and therefore $\frac{1}{\sqrt{np}\sigma_0}\sum_{i=1}^n(y_i - \Lambda\eta_{0i}) \overset{d}{=} \omega/\sqrt{p}$ where $\omega \sim \mathrm{N}_p(0, I_p)$. Then from Rudelson and Vershynin (2013, Theorem 2.1) for some $c > 0$ and any $\tau_{np} > 0$ $\mathbb{P}_0^p\phi_p = \Pr\left(\left|\frac{1}{\sqrt{p}}\|\omega\| - 1\right| > \tau_{np}\right) \leq 2\exp\left(-pc\tau_{np}^2\right)$. Since $\sum_{p=1}^\infty \mathbb{P}_0^p\phi_p < \infty$, by Borel-Cantelli lemma $\phi_p \to 0$ $\mathbb{P}_0^p$-a.s.

Notably when H_0 is not true i.e. under $\mathbb{P}_\vartheta^p$, $Y_i \overset{d}{=} \sigma\varphi_i + \Lambda\eta_i$ where $\varphi_i \overset{\text{iid}}{\sim} \mathrm{N}_p(0, I_p)$ for some $\vartheta \neq (\Lambda_0, \eta_0, \sigma_0)$ and therefore under $\mathbb{P}_\vartheta^p$

$$\mathbb{P}_\vartheta^p(1-\phi_p) \leq \Pr\left\{\frac{1}{\sqrt{pn}\sigma_0}\left\|\sum_{i=1}^n(\sigma\varphi_i + \Lambda\eta_i - \Lambda_0\eta_{0i})\right\| < 1 + \tau_{np}\right\}$$
$$\leq \Pr\left\{\frac{1}{\sqrt{pn}\sigma_0}\sum_{i=1}^n\|\Lambda\eta_i - \Lambda_0\eta_{0i}\| - 1 - \tau_{np} - \frac{\sigma}{\sigma_0} \leq \frac{\sigma}{\sigma_0}\left(\frac{1}{\sqrt{np}}\sum_{i=1}^n\|\varphi_i\| - 1\right)\right\}. \quad \text{(S.10)}$$

Notably for $\vartheta \in \tilde{\Theta}_p$, $\frac{1}{\sqrt{pn}\sigma_0}\sum_{i=1}^n\|\Lambda\eta_i - \Lambda_0\eta_{0i}\|$ is unbounded above for increasing p and $\frac{\sigma}{\sigma_0}$ is bounded thanks to (C3). Letting $C_p = \frac{1}{\sqrt{pn}\sigma_0}\sum_{i=1}^n\|\Lambda\eta_i - \Lambda_0\eta_{0i}\| - 1 - \tau_{np} - \frac{\sigma}{\sigma_0}$ we have $\liminf_{p\to\infty} C_p > 0$. Therefore, from Rudelson and Vershynin (2013, Theorem 2.1), we have for $\vartheta \in \tilde{\Theta}_p$, $\mathbb{P}_\vartheta^p(1-\phi_p) \leq 2\exp\left(-pncC_p^2\right)$. Hence the proof. ■

Theorem S.16 *Let* $\tilde{\Pi}_p$ *be the renormalized restriction of* Π *to the set* B_p^ϵ *defined in Theorem S.13. Then* $\mathbb{1}\{\bar{A}_p\} \to 0$ $\mathbb{P}_0^p$*-a.s.*

Proof If we can show that $\sum_{p=1}^\infty \mathbb{P}_0^p(\bar{A}_p) < \infty$, then by Borel-Cantelli lemma $\mathbb{P}_0^p[\limsup \bar{A}_p] = 0$ and henceforth $\mathbb{1}\{\bar{A}_p\} \to 0$ $\mathbb{P}_0^p$-a.s. Now

$$\mathbb{P}_0^p(\bar{A}_p) = \mathbb{P}_0^p\left[\frac{1}{p}\int\sum_{i=1}^n\left\{\frac{1}{\sigma^2}\|y_i - \mu_i\|^2 - \frac{1}{\sigma_0^2}\|y_i - \mu_{0i}\|^2 - \frac{1}{\sigma^2}\|\mu_i - \mu_{0i}\|^2 - p\left(\frac{\sigma_0^2}{\sigma^2} - 1\right)\right\}\mathrm{d}\tilde{\Pi}_p > 2\epsilon\right].$$

Notably under $\mathbb{P}_0^p$, $Y_i \overset{d}{=} \sigma_0\varphi_i + \mu_{0i}$ where $\varphi_i \overset{\text{iid}}{\sim} \mathrm{N}_p(0, I_p)$. Therefore

$$\mathbb{P}_0^p(\bar{A}_p) = \Pr\left[\frac{1}{p}\int\sum_{i=1}^n\left\{\left(\frac{\sigma_0^2}{\sigma^2} - 1\right)(\|\varphi_i\|^2 - p) + 2\frac{\sigma_0}{\sigma^2}\varphi_i^{\mathrm{T}}(\mu_i - \mu_{0i})\right\}\mathrm{d}\tilde{\Pi}_p > 2\tilde{\epsilon}\right]$$
$$\leq \Pr\left[\frac{1}{p}\sum_{i=1}^n(\|\varphi_i\|^2 - p)\int\left(\frac{\sigma_0^2}{\sigma^2} - 1\right)\mathrm{d}\tilde{\Pi}_p > \tilde{\epsilon}\right] +$$
$$\Pr\left[\frac{2}{p}\int\sum_{i=1}^n\left\{\frac{\sigma_0}{\sigma^2}\varphi_i^{\mathrm{T}}(\mu_i - \mu_{0i})\right\}\mathrm{d}\tilde{\Pi}_p > \tilde{\epsilon}\right]. \quad \text{(S.11)}$$

Let us consider the first term of (S.11). Notably

$$\Pr\left[\frac{1}{p}\sum_{i=1}^{n}(\|\varphi_i\|^2-p)\int\left(\frac{\sigma_0^2}{\sigma^2}-1\right)\mathrm{d}\tilde{\Pi}_p>\tilde{\epsilon}\right]\le\Pr\left[\frac{1}{p}\left|\sum_{i=1}^{n}(\|\varphi_i\|^2-p)\right|\int\left|\frac{\sigma_0^2}{\sigma^2}-1\right|\mathrm{d}\tilde{\Pi}_p>\tilde{\epsilon}\right]. \tag{S.12}$$

From (C3) we have that σ lies in a compact interval. Hence the integral in the right hand side of (S.12) is bounded above by some positive constant, say $C_{\sigma,1}$. Therefore,

$$\Pr\left[\frac{1}{p}\sum_{i=1}^{n}(\|\varphi_i\|^2-p)\int\left(\frac{\sigma_0^2}{\sigma^2}-1\right)\mathrm{d}\tilde{\Pi}_p>\tilde{\epsilon}\right]\le\Pr\left[\frac{1}{p}\left|\sum_{i=1}^{n}(\|\varphi_i\|^2-p)\right|>\frac{\tilde{\epsilon}}{C_{\sigma,1}}\right]\le 2e^{-pC_{\sigma,2}}$$

for some positive constant $C_{\sigma,2}>0$. The second inequality in the above equation follows from Rudelson and Vershynin (2013, Theorem 2.1). Clearly

$$\sum_{p=1}^{\infty}\Pr\left[\frac{1}{p}\sum_{i=1}^{n}(\|\varphi_i\|^2-p)\int\left(\frac{\sigma_0^2}{\sigma^2}-1\right)\mathrm{d}\tilde{\Pi}_p>\tilde{\epsilon}\right]<\infty. \tag{S.13}$$

Now we consider the second term of (S.11). As $\varphi_i=(\varphi_{i1},\ldots,\varphi_{ip})^{\mathrm{T}}$ (similarly μ_i and μ_{0i} are also p-dimensional vectors) we can write

$$\begin{aligned}\Pr\left[\frac{2}{p}\int\sum_{i=1}^{n}\left\{\frac{\sigma_0}{\sigma^2}\varphi_i^{\mathrm{T}}(\mu_i-\mu_{0i})\right\}\mathrm{d}\tilde{\Pi}_p>\tilde{\epsilon}\right]&=\Pr\left[\frac{2}{p}\sum_{i=1}^{n}\sum_{j=1}^{p}\varphi_{ij}\int\left\{\frac{\sigma_0}{\sigma^2}(\mu_{ij}-\mu_{0ij})\right\}\mathrm{d}\tilde{\Pi}_p>\tilde{\epsilon}\right]\\&\le\exp\left[-\frac{p^2\tilde{\epsilon}^2}{4\sigma_0^2\sum_{i=1}^{n}\sum_{j=1}^{p}E^2_{\tilde{\Pi}_p}\left\{\frac{1}{\sigma^2}(\mu_{ij}-\mu_{0ij})\right\}}\right],\end{aligned}$$

where $E_{\tilde{\Pi}_p}$ denotes the expectation with respect to the probability measure $\tilde{\Pi}_p$. The above inequality follows from sub-Gaussian concentration bounds. Now

$$\begin{aligned}\sum_{i=1}^{n}\sum_{j=1}^{p}E^2_{\tilde{\Pi}_p}\left\{\frac{1}{\sigma^2}(\mu_{ij}-\mu_{0ij})\right\}&\le\sum_{i=1}^{n}E_{\tilde{\Pi}_p}\frac{1}{\sigma^4}\|\mu_i-\mu_{0i}\|^2\ \text{(by Jensen's inequality)}\\&=E_{\tilde{\Pi}_p}\frac{1}{\sigma^4}\sum_{i=1}^{n}\times E_{\tilde{\Pi}_p}\|\mu_i-\mu_{0i}\|^2.\end{aligned} \tag{S.14}$$

Since we consider independent priors on σ,Λ and η_i, (S.14) follows from its preceding step. Note that on the set B_p^{ϵ}

$$n\log\frac{\sigma^2}{\sigma_0^2}+n\left(\frac{\sigma_0^2}{\sigma^2}-1\right)+\frac{1}{p\sigma^2}\sum_{i=1}^{n}\|\mu_i-\mu_{0i}\|^2<2\epsilon. \tag{S.15}$$

From the inequality $\log x<x-1$ we see that $n\log\frac{\sigma^2}{\sigma_0^2}+n\left(\frac{\sigma_0^2}{\sigma^2}-1\right)>0$. Therefore for $\vartheta\in B_p^{\epsilon}$, in conjunction of (S.15) and (C3) we have $\frac{1}{p}\sum_{i=1}^{n}\|\mu_i-\mu_{0i}\|^2<2\epsilon\sigma_U^2\Rightarrow\frac{1}{p}\sum_{i=1}^{n}E_{\tilde{\Pi}_p}\|\mu_i-\mu_{0i}\|^2<$

$2\epsilon\sigma_U^2$. Also thanks to (C3) $E_{\tilde{\Pi}_p}\frac{1}{\sigma^4}$ is bounded above. Hence the term in (S.14) is bounded above and consequently

$$\sum_{p=1}^{\infty} \Pr\left[\frac{2}{p}\int \sum_{i=1}^{n}\left\{\frac{\sigma_0}{\sigma^2}\varphi_i^{\mathrm{T}}(\mu_i-\mu_{0i})\right\} d\tilde{\Pi}_p > \tilde{\epsilon}\right] < \infty. \tag{S.16}$$

Combining (S.13) and (S.16) we conclude that $\sum_{p=1}^{\infty}\mathbb{P}_0^p(\bar{A}_p) < \infty$. Hence the proof. ■

S.3. Details on Simulation Studies

In this section, we discuss the data-generation strategies in three simulation scenarios: [1] Lamb, [2] mixture of sparse factor analyzers (MFA), and [3] mixture of log transformed zero inflated Poisson counts (SpCount) considered in Section 5 of the main manuscript. The observed p-dimensional data are $y_1,\dots,y_n$, k_0 is the number mixture components in the simulation truth and $\pi_1,\dots,\pi_{k_0}$ are the mixture probabilities attached to each cluster such that $\sum_{h=1}^{k_0}\pi_h = 1$.

Lamb We let the observed data

$$y_i = \Lambda\eta_i + \epsilon_i, \quad \eta_i \overset{\text{iid}}{\sim} \textstyle\sum_{h=1}^{k_0}\pi_h \mathrm{N}_d(\mu_h,\Delta_h), \quad \epsilon_i \overset{\text{iid}}{\sim} \mathrm{N}_p(0,\Sigma),$$

where Λ is a $p\times d$ order sparse matrix with many entries equal to zero, $\mu_h\in\mathbb{R}^d$, Δ_h is a $d\times d$ positive definite matrix, for all $h=1,\dots,k_0$ and Σ is a $p\times p$ order diagonal matrix with positive entries.

Mixture of sparse factor analyzers (MFA) We let the observed data

$$y_i \overset{\text{iid}}{\sim} \textstyle\sum_{h=1}^{k_0}\pi_h \mathrm{N}_p(\mu_h, \Lambda_h\Lambda_h^{\mathrm{T}} + \Sigma_h),$$

where Λ_h is a $p\times d$ order sparse matrix with many entries equal to zero, Σ_h is a $p\times p$ diagonal matrix with positive entries and $\mu_h\in\mathbb{R}^p$, for all $h=1,\dots,k_0$.

Mixture of log transformed zero inflated sparse Poisson counts (SpCount) Let $\{\ell_1,\dots,\ell_p\}$ be a random permutation of $1,\dots,p$, $r=\lfloor p/k_0\rfloor$ and define the set $S_h=\{\ell_{(h-1)\times r+1},\dots,\ell_{h\times r}\}$ for all $h=1,\dots,k_0$. Thus $\{S_1,\dots,S_{k_0}\}$ can be regarded as a random partition of $\{1,\dots,p\}$ where each partition has r elements. Additionally fix k_0 positive constants $\lambda_1,\dots,\lambda_{k_0}$, and let

$$w_{ij} \mid c_i = h \overset{\text{iid}}{\sim} \begin{cases} \mathrm{Pois}(\lambda_h) + \mathrm{N}(0,1) \text{ for all } j\in S_h, \\ 0 \text{ with probability 1 for all } j\notin S_h, \end{cases}$$

$$\Pr(c_i = h) = \pi_h \text{ for all } h=1,\dots,k_0.$$

where $\mathrm{Pois}(\lambda)$ is the Poisson distribution with mean λ and set $y_{ij}=\log(w_{ij}+1)$ for all $j=1,\dots,p$ and $i=1,\dots,n$. Thus the observed data y_i's are highly non-Gaussian within each cluster.

S.4. Additional Simulation Studies

S.4.1 Illustration of the Degenerate Clustering Behaviour

To show the degenerate clustering behavior discussed in Section 2 we performed two simple simulation experiments under the settings of Corollaries 2 and 3.

In the first experiment, we generate data from a five-component mixture model. Specifically, we assumed five well-separated Gaussians with equal proportions. The location vector for the h-th component is $\theta_h \mathbf{1}_p$ with $\mathbf{1}_p$ a p-dimensional vector of ones, $\theta_h \in \mathbb{R}$ and the values of θ_h ranging from -10 to +10. Each mixture component has identity covariance matrix. We fix $n = 10$ and $p = 20$. The left panel of Figure S.1 displays the distribution of the posterior median number of clusters in 100 replicates for a standard DP location mixture with hyperparameter specification satisfying Corollary 2 and proposed Lamb. For the DPM, we use the implementation in the `BNPmix` package (Corradin et al., 2021). Despite coming from a five-component mixture model, the data are grouped into a single cluster for most of the simulation replicates under the DPM specification, consistent with the limiting behavior described by Corollary 2.

In the second experiment, we assume a single p-variate normal distribution with mean zero, and identity covariance. As before, we fix $n = 10$ and $p = 20$. The results obtained assuming a DP mixture with the hyperparameter specification satisfying Corollary 3 and the proposed Lamb are reported in the right panel of Figure S.1. These results clearly show that the limiting behavior described by Corollary 3 is evident already for the moderate $p = 20$. Notably, the proposed Lamb avoids these pitfalls and is associated to a median number of clusters that is centered around the true values.

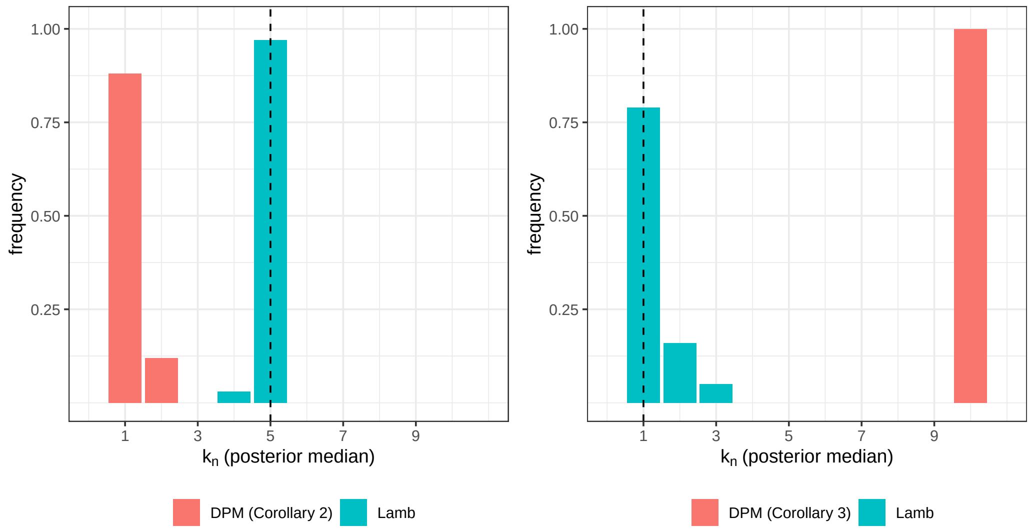

Figure S.1: Empirical distribution of the posterior median number of clusters in 100 replicates under the first (left) and second (right) simulation experiment. DPM hyperparameter specification satisfies Corollary 2 (left) and Corollary 3 (right). Vertical dashed lines represent the true number of clusters.

S.4.2 Recovering the Latent Space

To empirically illustrate the robustness of assumptions (C1) and (C2) used to prove the theory of Section 4, we perform a simple simulation study. These conditions ensure that the data contain increasing information on the latent factors as p increases. Increasing p means that we observe

additional y_{ij} variables for each subject. Each of these variables can have very small correlation with the latent factor η_{0i} and there will still be a build-up of information.

To see this, we generate random y_i for $i = 1, \dots, 4$ and $p \in \{20, 200, 1000\}$. Data are generated as $y_i = \Lambda_0 \eta_{0i} + \epsilon_i$ where the factor loadings Λ_0's are generated according to

$$\lambda_{0jh} \overset{\text{iid}}{\sim} \pi \delta_{\{0\}} + (1-\pi)\delta_{\{0.5\}},$$

where $\delta_{\{a\}}$ denotes a Dirac's delta mass at value a. The true latent factors are simulated as $\eta_{0i,j} \sim \mathrm{N}(i + j - 1, 0.05^2)$ where $\eta_{0i} = (\eta_{0i1}, \dots, \eta_{0id})^{\mathrm{T}}$. We consider two error distributions ensuring low signal-to-noise ratio, and specifically $\epsilon_{ij} \sim \mathrm{N}(0, 25)$ and $\epsilon_{ij} \sim t_3$ where t_3 denotes a central t distribution with 3 degrees of freedom. We set $\pi = 0.2$ and the latent dimension $d = 2$.

To examine the level of recovery, for the m-th MCMC iteration, we regress the true factors with their current value in the m-th iteration. Specifically we stack all η_{0ij} across $i = 1, \dots, n$ in a vector and use it as response variable, while using as predictor the vector containing all $\eta_{ij}^{(m)}$ of the m-th iteration. We do this for each iteration after the burn-in.

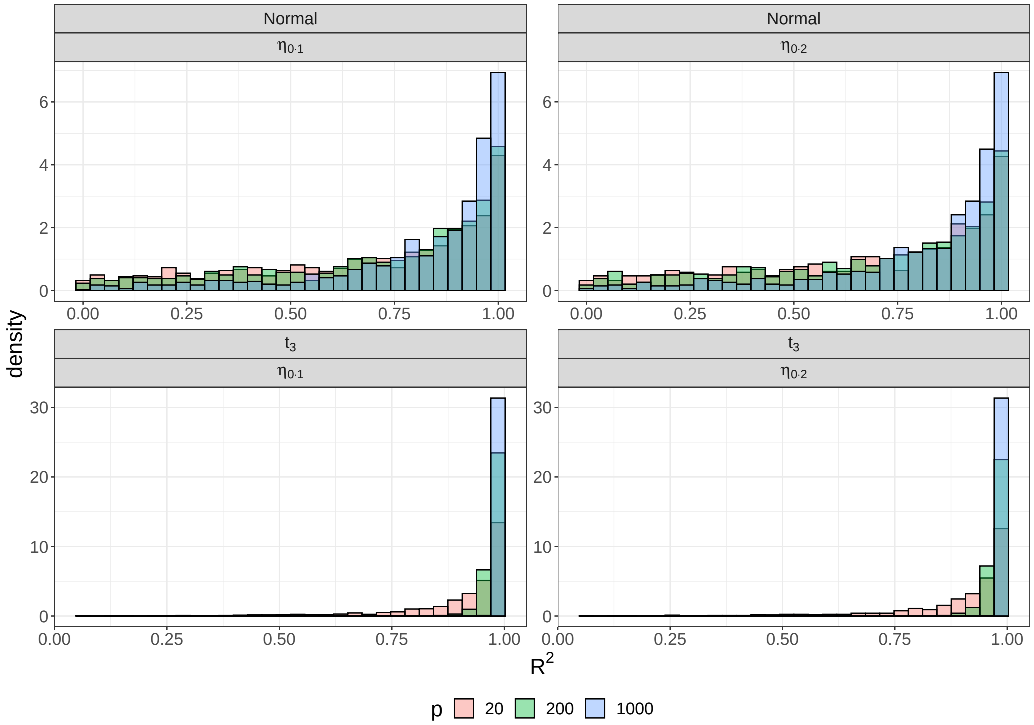

Figure S.2: Posterior distributions of the coefficient of determination R^2 of the linear regressions of the true latent factors on the associated posterior samples for two error distributions ($\mathrm{N}(0, 25)$, in the upper quadrants and t_3, in the bottom quadrants). The different dimensions p are denoted by the different colors.

Clearly, the latent factors are non-identifiable due to the well known rotational ambiguity and thus they can be learned only up to some non-singular matrix multiplication. Hence, to quantify

the accuracy in recovering the latent space, we consider the coefficient of determination R^2 of each fitted regression which is invariant of such identifiability issues. Figure S.2 reports the results. For both error distributions under consideration, as p grows the posterior distributions of the coefficients of determination concentrate near one implying that with more variables we improve on the learning of the latent space even with low signal-to-noise ratios.

S.4.3 Small Sample Studies

In this section, we do additional simulation studies. We consider the same setups considered in Section 5 of the main paper but take the sample size $n = 500$. The true number of clusters is fixed to $k_0 \in \{10, 15\}$. The results depicted in Figure S.3 are overall consistent with those reported in Section 5.

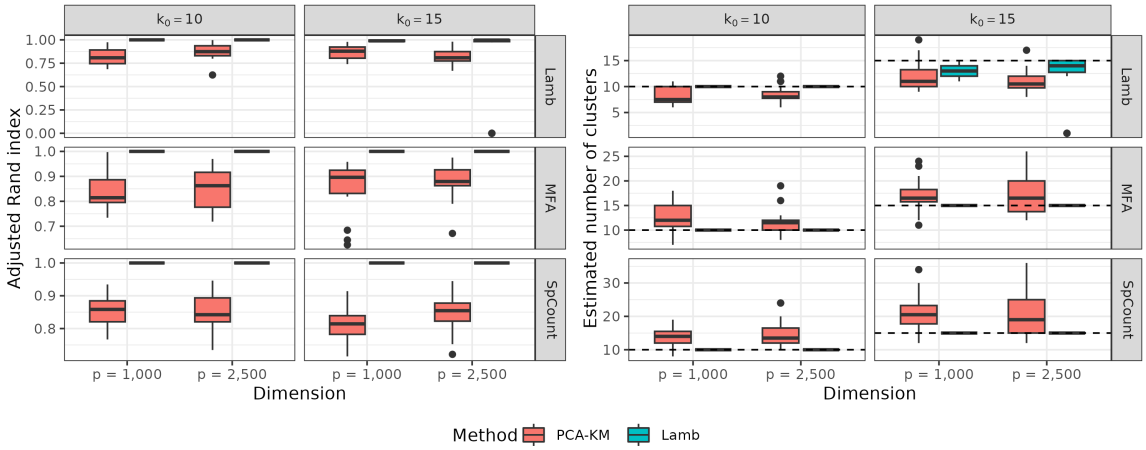

Figure S.3: Comparison between our proposed Lamb and the two-stage PCA-KM approach: Distributions of the adjusted Rand indices (left plot) and estimated number of clusters (right plot) in 20 replicated experiments. Horizontal dashed lines denote the true number of clusters. The true simulation scenarios, reported in each row, are labeled as Lamb for the model of Section 3, MFA for mixture of factor analyzers and SpCount for the log transformed zero inflated sparse Poisson counts.

S.4.4 Figures Associated to Section 5

Figures S.4-S.9 report the UMAP (McInnes et al., 2018) plots of the simulated datasets of Section 5, corresponding to the replicate with median adjusted Rand index (Rand, 1971). In each figure, the upper and lower panels show the true clustering and the estimated clustering obtained by the Lamb model, respectively. Each figure's caption specifies the true number of clusters (k_0) and the dimension (p).

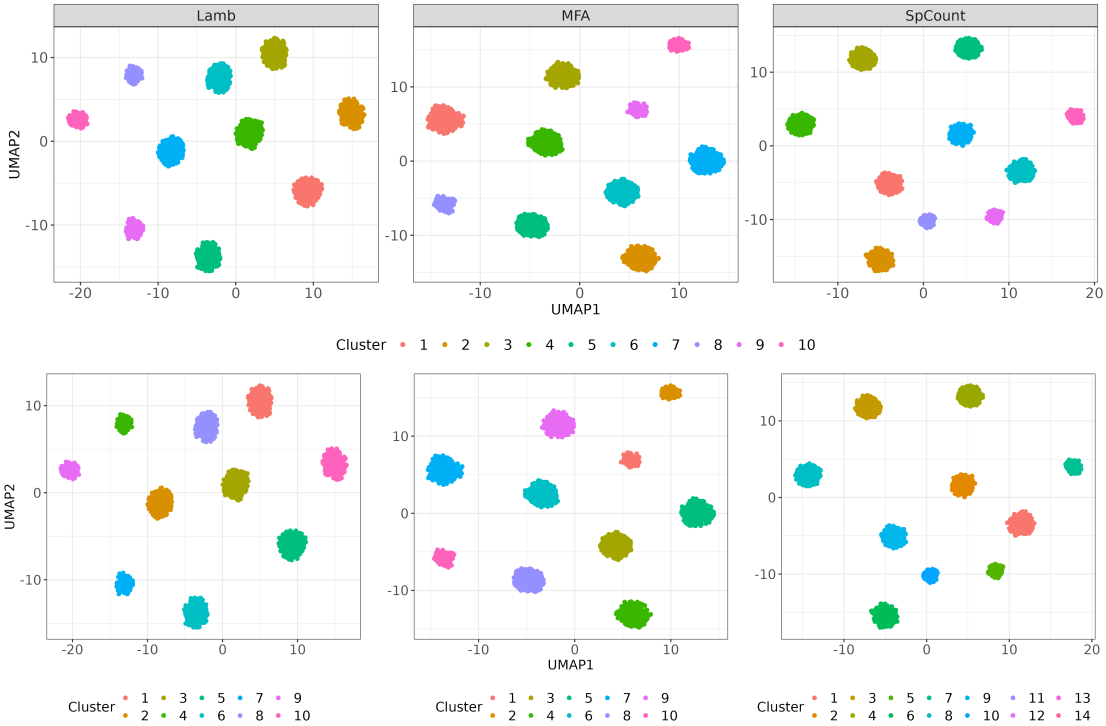

Figure S.4: $k_0 = 10$, $p = 1,000$.

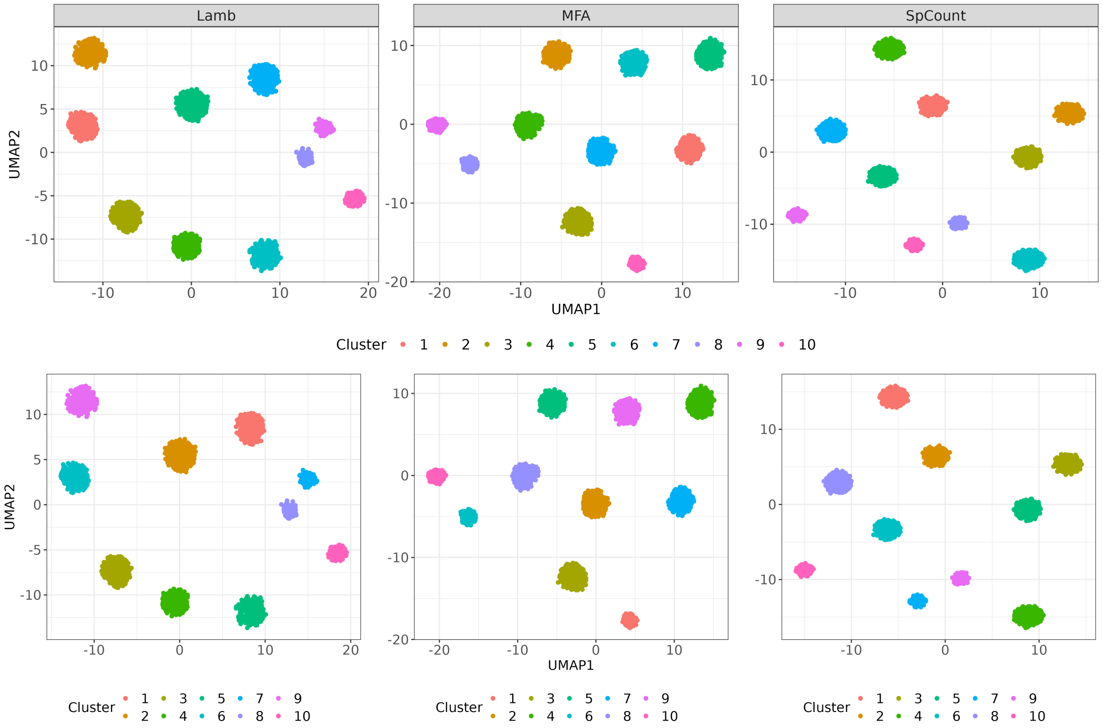

Figure S.5: $k_0 = 10$, $p = 2,500$

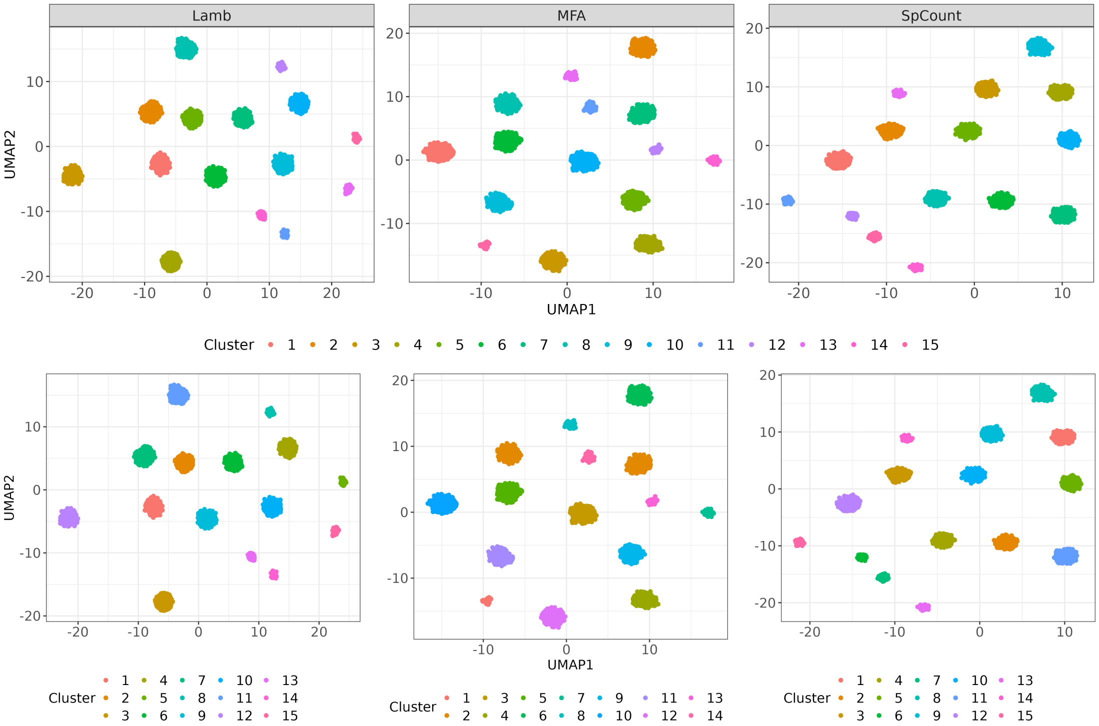

Figure S.6: $k_0 = 15$, $p = 1,000$

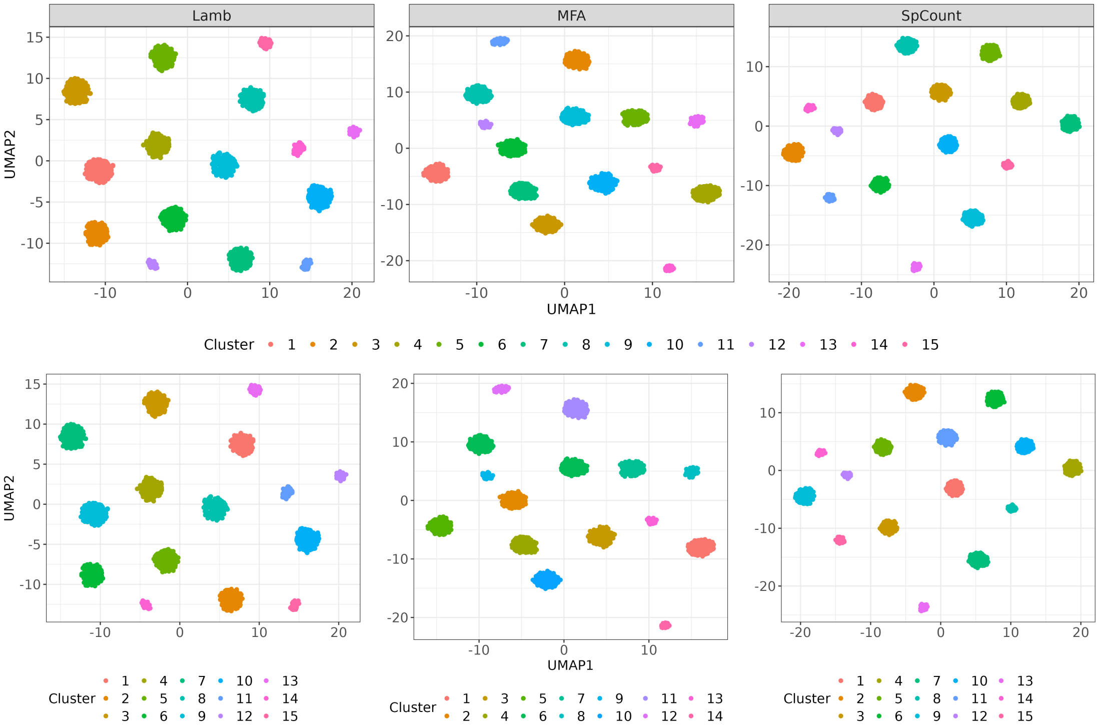

Figure S.7: $k_0 = 15$, $p = 2,500$

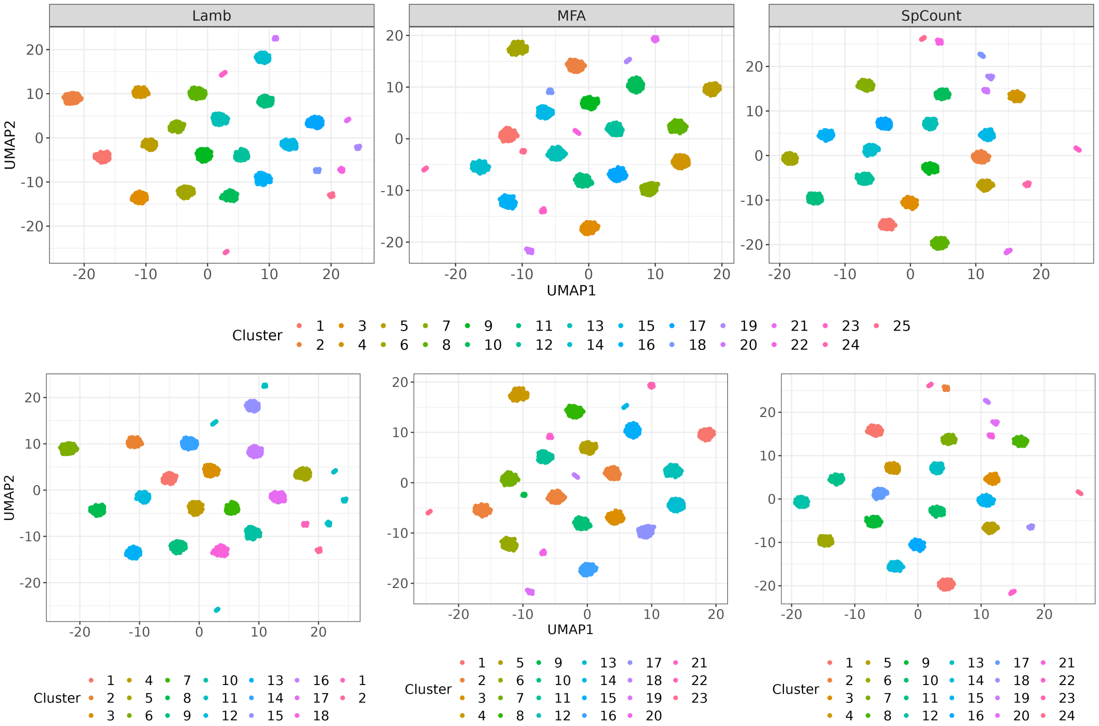

Figure S.8: $k_0 = 25$, $p = 1,000$

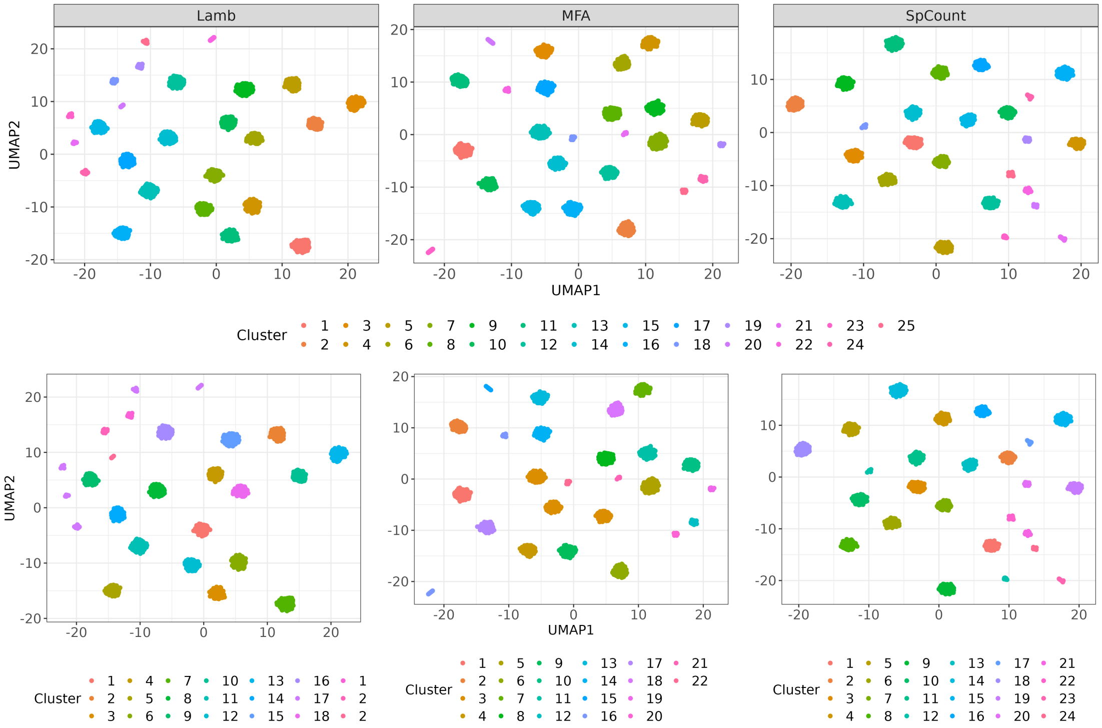

Figure S.9: $k_0 = 25$, $p = 2,500$

S.5. MCMC Convergence Diagnostics in the Cell Line Application

In this section, we provide convergence diagnostics of the MCMC sampler discussed in Section 3.2. Note that most of the variables that are sampled are latent objects and not identifiable. Hence we compute the $\log$-likelihood of $y_{1:n} \mid \Lambda, \eta, \Sigma$ across the MCMC samples. On these $\log$-likelihoods, we show traceplots and Geweke convergence diagnostics (Geweke, 1992) as implemented in the `coda R` package (Plummer et al., 2006). The results are shown in Figure S.10 and they indicate evidence towards good mixing.

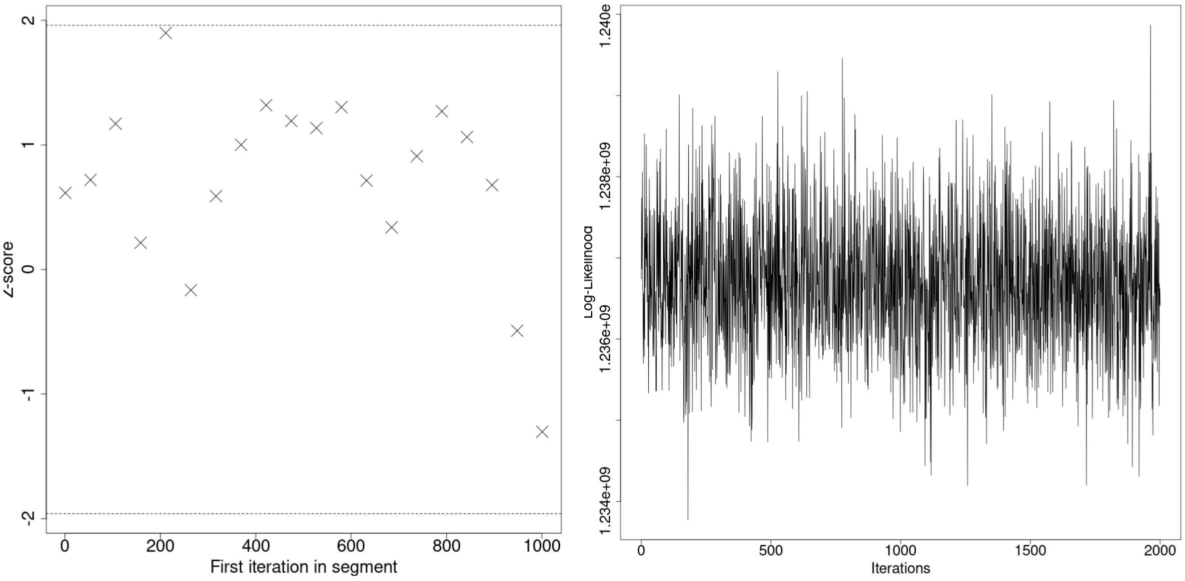

Figure S.10: MCMC Convergence Diagnostics in the Cell Line Application: The joint $\log$-likelihoods of the $y_{1:n} \mid \Lambda, \eta, \Sigma$ are first calculated across the MCMC iterations. The Geweke convergence diagnostic on the $\log$-likelihoods is shown in the left panel and their traceplot in the right panel.
